# Chromosomal inversions modulate 3D chromatin interactions and regulatory architecture in *Anopheles gambiae*

**DOI:** 10.21203/rs.3.rs-9213027/v2

**Published:** 2026-08-13

**Authors:** Kathryn S. Taquet, Cameron E. Anderson, Kenneth D. Vernick, Michelle M. Riehle

**Affiliations:** 1 Department of Microbiology and Immunology, Medical College of Wisconsin, Milwaukee, WI 53226; 2 Institut Pasteur, Université Paris Cité, Arboviruses and Insect Vectors Unit, Paris, France

**Keywords:** Anopheles, gene regulation, proximity-ligation, Micro-C, inversion biology

## Abstract

**Background:**

Malaria parasite transmission by *Anopheles* mosquitoes remains a worldwide health burden, and understanding factors underlying natural vector susceptibility would inform rational design of vector control. The 2La chromosomal inversion segregates in the major vectors of human malaria, *Anopheles coluzzii* and *Anopheles gambiae,* and is associated with natural variation for malaria susceptibility, though underlying mechanisms are unknown. Here, we characterize alterations of chromatin conformation and gene expression induced by the two 2La inversion allelic forms, the ancestral 2La and the derived 2L+^a^ forms as well as by the two 2Rbc inversion allelic forms. We employ several novel applications of proximity ligation sequencing to refine the mosquito regulatory genome to a new level of resolution.

**Results:**

We analyzed the 2La inversion breakpoints in *A. coluzzii* hemocyte-like cell lines for the allelic 2La inversion karyotypes. Utilizing a novel combination of Micro-C, bulk RNA-sequencing, and ATAC-sequencing, our results detected transcriptional enhancers and genes that are reciprocally rewired by the physical rearrangement caused by the inversion. Similar analyses on the 2Rbc inversion revealed enhancer rewiring without reciprocity between alternate inversion forms, likely due to the distinct evolutionary histories between the 2La and 2Rbc inversions. Through development and application of novel distance-normalized interaction frequency analysis on Micro-C data, we identify a novel common enhancer for the APL1 gene family and a novel candidate enhancer for LRIM1, all of which are major parasite antagonist immune genes within the 2La inversion.

**Conclusions:**

This multifaceted approach yields high resolution characterization of gene *cis*-regulation within *Anopheles* mosquitoes, and specifically within the context of a malaria-associated paracentric inversion. Additionally, development of analytical methods for proximity ligation data allows fine scale exploration of mosquito chromatin interactions and are broadly applicable across species. Finally, the newly identified regulatory architecture of an inversion associated with *Plasmodium* infection, and of multiple parasite-antagonistic immune genes, highlights candidates for potential further development of a novel vector control toolkit.

## BACKGROUND

The *Anopheles gambiae* species complex contains the primary vectors of malaria, a disease caused by the *Plasmodium* parasite that continues to be a major worldwide health burden. Despite the implementation of widespread vector control strategies over 20 years ago, including insecticide-treated nets and indoor residual spraying, malaria still causes over 600,000 death per year – mainly in Sub-Saharan Africa^1^. Recent rising malaria case rates are the cumulative result of increased drug and insecticide resistance within the parasite and mosquito populations, respectively^1, 2^. Efforts to understand the mechanisms underlying natural variation in mosquito susceptibility to *Plasmodium* infection would inform rational design of vector control tools. While the mosquito immune response to parasite infection has been well studied, the focus has largely been on candidate immune factors including the APL1 gene family^3–8^ ((APL1A (=AGAP007036); APL1B (=AGAP007035); (APL1=AGAP007033), all on chromosome arm 2L within the 2La inversion), TEP1^3–5, 9^ (AGAP010815, on chromosome 3L), LRIM1^3–6, 10–13^ (AGAP006348, on chromosome arm 2L within the 2La inversion), and CEC1^13–15^ (AGAP000693, on chromosome X), and very little is known about how these antagonists of malaria infection are regulated. Recent work has begun to explore the role of regulatory elements and chromatin accessibility in response to parasite infection^16, 17^. However, as regulatory elements can be located at significant distance from their target gene(s), a gap remains in linking regulatory elements with target genes. It is only with proximity ligation sequencing, or similar techniques providing insight into 3D chromatin folding, that *Anopheles* regulatory biology can be further refined.

One form of genetic variation common to dipterans and prevalent across *Anopheles* species is the chromosomal inversion^18–21^. Primary vectors of malaria, *Anopheles coluzzii* and *Anopheles gambiae,* are polymorphic for the 2La inversion, a monophyletic 21.6MB paracentric chromosomal inversion on the 2L chromosome arm^22^, whose breakpoint positions are 20,528,072bp and 42,165,626bp^23^. The 2La inversion, whose allelic forms are denoted as 2La (ancestral) and 2L+^a^ (derived)^22, 24^, is associated with an intrinsic differential resistance to *P. falciparum* infection in Africa, in which 2L+^a^ mosquitos are more susceptible to *Plasmodium* infection^25, 26^. Additionally, the 2La inversion is associated with a number of other extrinsic vector competence factors, including differences in mosquito resting and biting behavior^25, 27^, aridity^18, 28^ and thermal tolerance^28–30^, as well as insecticide resistance^31^. There are also several known inversions on the 2R chromosome arm that segregate in A. coluzzii and A. gambiae: 2Rj, 2Rb, 2Rc, 2Ru, and 2Rd, which are associated with environmental adaptations^32–35^. Unlike the monophyletic 2La inversion^25^, 2R inversions involve breakpoint reuse, resulting in the 2Rb and 2Rc inversions occurring directly adjacent to each other and being in strong linkage disequilibrium as well as the dual emergence of the 2R+^b^ inversion karyotype^32, 36, 37^. Mechanisms underlying the 2La inversion-associated phenotypes related to vector competence and environmental adaptation phenotypes associated with 2R inversions are not fully understood, but differences in chromatin conformation due to physical rearrangement of the chromosome likely play a role, particularly at inversion breakpoints where novel chromosome junctions were created.

The hierarchy of chromatin folding includes, in ascending order from more local to more macro, chromatin loops which can bring enhancers (non-coding regulatory elements) into close proximity to promoters, enabling modulation of target gene(s) transcription; topologically-associating domains (TADs), which can be described as regulatory neighborhoods wherein enhancers are limited to acting on promoters located within the same TAD; and finally, A/B compartments, which are regions of active and inactive chromatin, respectively^38, 39^. Chromatin conformation capture techniques, including 3C, 4C, and Hi-C/Micro-C, involve crosslinking chromatin within the nucleus, thus capturing the chromatin in its folded state, followed by fragmentation and proximity-ligation to enable identification of the interacting chromatin regions^40^. The different chromatin conformation capture techniques vary in their breadth of genomic coverage, ranging from locus-specific (e.g. 3C) to unbiased genome-wide (e.g. Hi-C) interrogation^40^. Micro-C, a variation of Hi-C that uses the unbiased cleaver micrococcal nuclease (MNase) for digestion instead of restriction enzymes thus enabling finer resolution, is a comprehensive and unbiased approach for determining chromatin conformation. Following fragmentation of crosslinked chromatin, the fragmented chromatin ends are repaired and biotinylated, proximity-ligation is performed, and the biotinylated DNA undergoes high-throughput paired-end sequencing. Therefore, chromatin interactions are queried on a genome-wide scale.

Broad-scale analysis of Hi-C experimental data in *Anopheles* has been used to facilitate chromosome/genome assembly^41–44^, inversion breakpoint mapping^36^, and TAD structure determination^42^ providing researchers with some understanding of the chromatin organization within *Anopheles* mosquitoes, but much remains unknown. Genome-wide maps of enhancers and chromatin accessibility in *Anopheles* have been generated by us using self-transcribing active regulatory region sequencing (STARR-seq) and assay for transposase-accessible chromatin with high-throughput sequencing (ATAC-seq) approaches, respectively. Additionally, well-annotated genomes (PEST version AgamP4 for the 2L+^a^ inversion form^45^ and MOPTI version 2021-03-25 for the 2La inversion form^41^) are available for *A. gambiae* and *A. coluzzii*, respectively. The integration of genomic datasets describing candidate regulatory elements and chromatin accessibility with proximity ligation sequencing enables fine-scale exploration of chromatin interactions, including the identification of enhancer-promoter interactions, which has been achieved in other organisms^46–48^.

Here, we perform Micro-C, bulk RNA-sequencing, and ATAC-sequencing on *A. coluzzii* hemocyte-like cells fixed for alternate forms of the large paracentric 2La inversion to characterize inversion-associated changes in chromatin conformation. With the availability of genome-wide datasets of genes^45^, candidate enhancers^49^, and regions of open chromatin^16, 17, 50, 51^ coupled with the generation of proximity ligation sequencing data, we explore enhancer rewiring associated with the 2La and 2Rbc inversions, which have distinct evolutionary histories, and prioritize candidate regulatory elements for two Plasmodium antagonists located on the 2L chromosome arm, APL1 and LRIM1. Our multifaceted approach integrating multi-omics datasets enables high resolution exploration of chromatin architecture particularly in the context of chromosomal inversions which are common across Dipterans and associated with extensive phenotypic plasticity.

## RESULTS

### Micro-C and bulk RNA-sequencing of alternate 2La inversion karyotypes

Hemocytes are innate immune cells that respond to infection with pathogens, including *Plasmodium*, and mediate different immune effector mechanisms, such as phagocytosis, lysis, and melanization^52^. Two hemocyte-like *A. coluzzii* cell lines Sua4.0 (cell line homozygous for the 2La karyotype, will be referred to as 2La cells throughout)^53, 54^ and Ag55 (cell line homozygous for the 2L+^a^ karyotype, will be referred to as 2L+^a^ cells throughout)^54–56^ were selected for their known alternate 2La inversion karyotypes to investigate the effects the 21.6MB 2La inversion, which is associated with *Plasmodium* susceptibility^25, 26^, has on chromatin conformation and gene expression. We utilize hemocyte-like cell lines as a convenient model for genomic analysis and given the role of hemocytes in mosquito innate immunity and the response to *P. falciparum* infection but acknowledge that results do not necessarily reflect the chromatin interactions or behavior of genes in different cell and tissue types of the whole mosquito.

Micro-C and bulk RNA-sequencing libraries were generated from *A. coluzzii* hemocyte-like cells confirmed to be homozygous for the ancestral (2La) or derived (2L+^a^) form of the large 2La inversion using a previously published molecular karyotyping assay^23^ (Sua4.0 = 2La/2La^53, 54^; Ag55 = 2L+^a^/2L+^a54–56^). Raw reads for both cell line samples were mapped to PEST (2L+^a^) reference genome version AgamP4 (VectorBase release 68) to facilitate sample comparison. PEST was the reference genome of choice because it is the most mature chromosome-level Anopheline genome assembly^41, 45^. When 2L+^a^/2L+^a^ samples are mapped to PEST (2L+^a^ reference genome), the resulting contact matrix of chromosome arm 2L shows the classical pattern of a bright diagonal of nearby interactions (Fig1A). Generally, when a sample of one inversion karyotype is mapped to a reference genome of the opposite inversion karyotype, the regions near the two inversion breakpoints that interact appear as a butterfly contact pattern^21, 57^. Therefore, conversely, when 2La/2La samples are mapped to the 2L+^a^ reference genome, a butterfly pattern emerges in chromosome arm 2L at ~20.5MB interacting with ~42MB, indicative of the 2La chromosomal inversion whose breakpoint positions are 20,528,072bp and 42,165,626bp^23^ (Fig1B).

Ag55 (2L+^a^ sample) contact matrices of chromosome arm 2R revealed an irregular interaction structure, marked by the asterisks in Figure 1C. Approximate PEST positions of the three points of interactions are 19MB interacting with 26.6MB (consistent with the 2Rb inversion whose breakpoint positions are 19,023,925bp and 26,758,676bp^32^), 26.6MB interacting with 32MB (consistent with the 2Rc inversion whose breakpoint positions are 26,758,676bp and 31,488,544bp^36^), and 19MB interacting with 32MB (which is likely due to the two inverted regions being directly adjacent). This irregular interaction structure is in Ag55 samples (Fig1C) but not Sua4.0 samples (Fig1D), and it is present when mapping Ag55 samples to either PEST (Fig1C) or MOPTI (Fig1E) reference genomes, indicating that PEST and MOPTI are colinear for 2R+^b^ and 2R+^c^ which is consistent with the literature^41^. Other known inversions on chromosome arm 2R are 2Rj^58^, 2Ru^42^, and 2Rd^59^, whose PEST breakpoint coordinates are 3,262,186-15,750,717bp, 31,102,383-35,322,826bp, and 31,495,381-42,375,004bp, respectively. Both cell lines and both reference genomes are 2R+^jud^/2R+^jud^, evidenced by the lack of irregular interaction structure near 2Rj, 2Ru, and 2Rd breakpoint coordinates (Fig1C–F). We further investigated the Micro-C findings with the highest quality molecular karyotyping assays for 2Rb^33^, 2Rc^37^, 2Rj^60^, and 2Ru^61^on Ag55 and Sua4.0 cells. Ag55 was confirmed to be 2R+^j^bc+^u^/2R+^j^bc+^u^, and Sua4.0 was confirmed to be 2R+^jbcu^/2R+^jbcu^ (Additional File 1, FigS1). The 2Rc inversion has been reported to be in almost perfect linkage disequilibrium with the inverted form of either 2Rb or 2Ru^37^, which is consistent with our cell line karyotypes. Contact matrices of Ag55 and Sua4.0 replicates mapped to PEST and MOPTI for all chromosomes are in Additional File 1, FigS2.

Using Hi-C/Micro-C contact matrices, multiHiCcompare^62^ identifies differentially interacting regions between samples, regardless of distance between the interacting regions. The differential interactions that appear to span the entire length of the 2La inversion when 2La samples are mapped to PEST (2L+^a^ reference genome) are an artifact of mapping to a reference genome of alternative inversion karyotype and provide quality control evidence that Micro-C of our 2La and 2L+^a^ samples was successful (Fig1G). Assessing differential interactions, multiHiCcompare^62^ identified 28,273 significant differentially interacting regions (DIRs) between 2La cells and 2L+^a^ cells on chromosome arm 2L (10,508 are more frequent in 2La/2La cells and 17,765 are more frequent in 2L+^a^/2L+^a^ cells). DIRs are enriched over the 2La inversion where 18,861 (~66.7%) of the differential interactions have at least one anchor within the 21.6MB 2La inversion (~43.8% of the 2L chromosome arm) (p<0.0001). Comparatively for chromosome arm 2R which contains the 2Rbc inversion, another inversion karyotype that the cell line samples are differentially fixed for, multiHiCcompare identified 26,075 significant DIRs between 2Rbc cells and 2R+^bc^ cells (11,298 are more frequent in 2Rbc/2Rbc cells and 14,777 are more frequent in 2R+^bc^/2R+^bc^ cells). Unlike with the 2La inversion, DIRs are not enriched over the 2Rbc inversion (p=0.45): 4,442 (~17.0%) of the differential interactions have at least one anchor within the 12.4MB 2Rbc inversion (~20.3% of the 2R chromosome arm). These differing results are not surprising given the distinct evolutionary histories for the 2La and 2Rbc inversions with the 2La inversion having a monophyletic origin^22, 25^ contracting with breakpoint re-use with 2R inversions^36^. The anchor regions of significantly frequent or infrequent interactions are plotted in Additional File 1, FigS3.

To assess differential gene expression, DESeq2^63^ analysis identified 2,297 differentially expressed genes (DEGs) between 2La and 2L+^a^ cells genome-wide with 477 of the DEGs on chromosome arm 2L and 591 DEGs on chromosome arm 2R. Unlike with DIRs within the 2La inversion, there is no enrichment of DEGs over the 2La inversion (p=0.69): 220 (~46.1%) of the DEGs on the 2L chromosome arm lie within the 2La inversion and 257 (~53.9%) of the 2L DEGs are outside the 2La inversion. There is also no enrichment of DEGs over the 2Rbc inversion (p=0.62): 130 (~22.0%) of the DEGs on the 2R chromosome arm are within the 2Rbc inversion and 461 (~78.0%) of the 2R DEGs are outside the 2Rbc inversion. Visualization of the distribution of significantly up- and down-regulated genes can be found in Additional File 1, FigS3. Although genetic variation between inversion forms is highest nearest inversion breakpoints^30^, gene expression changes measured by RNA-seq are the cumulative result from the cascades of gene expression networks. A list of all DIRs with fold change and adjusted p-values can be found in Additional File 3, Table S1. A list of all DEGs with their gene description, fold change, and adjusted p-values can be found in Additional File 3, Table S2.

### Evidence of novel enhancer rewiring associated with the 2La chromosomal inversion

One consequence of chromosomal inversions is that they can seed the process of enhancer rewiring - the evolution of novel interactions between enhancers and their target genes due to the change in physical proximity driven by the inversion^46, 64–66^. To better understand the impact that chromatin conformation changes resulting from the 2La inversion have on gene expression, a focused analysis over the 2La breakpoints was performed, coupling Micro-C proximity ligation sequencing and bulk RNA sequencing data. Enhancers were defined as being detected in at least 2 of 3 replicates from a previously published STARR-seq experiment^49^ (Additional File 2). This includes 788 previously published STARR-seq identified enhancers on the 2L chromosome arm detected in all 3 replicates (3,288 genome wide enhancers) as well as 274 additional STARR-seq identified enhancers on the 2L chromosome arm that were detected in 2 of 3 replicates (1,237 genome wide) for a total of 1,062 STARR-seq candidate enhancers across the 2L chromosome arm. Promoters were defined as the 1000bp region upstream of the protein coding gene as annotated in VEuPathDB^67, 68^. These definitions of enhancers and promoters are used throughout all analyses. To identify evidence of novel enhancer rewiring associated with the 2La chromosomal inversion, three filters were applied to the 28,273 differential interactions between 2La and 2L+^a^ samples identified via multiHiCcompare^62^: 1) the interaction had to occur over the proximal or distal breakpoint of the 2La inversion, 2) one anchor of the interaction had to contain at least one enhancer while the other anchor had to contain the promoter region of at least one DEG, and 3) the enhancers and promoter regions of the DEGs must interact over a 2La breakpoint in both 2La and 2L+^a^ cell line samples (Fig2A). Thirteen interactions met all three criteria, and they contained enhancers EP1, EP2 (located just outside of the proximal breakpoint), ED1, ED2, and ED3 (located just outside the distal breakpoint) and genes AGAP005781 (located inside the inversion on the proximal end in PEST), AGAP007066, AGAP007064, and AGAP007063 (located inside the inversion on the distal end in PEST), which have broad functions and may directly or indirectly influence known 2La inversion associated phenotypes (Fig2B). AGAP005781 is a glycine C-acetyltransferase, AGAP007066 is a tRNA-specific adenosine deaminase, AGAP007064 is an unspecified product, and AGAP007063 is origin recognition complex subunit 4^67, 68^. A complete table of gene name and function can be found in Additional File 3, Table S3. Near the proximal end of the inversion, in the cell line homozygous for the 2La ancestral form, EP1 and EP2 interact with AGAP007066, and EP2 additionally interacts with AGAP007064 and AGAP007063 while in the cell line homozygous for the 2L+^a^ derived form, EP1 and EP2 interact with AGAP005781. Near the distal end of the inversion, in the cell line homozygous for the 2La ancestral form, ED1, ED2, and ED3 interact with AGAP005781 while in the cell line homozygous for the 2L+^a^ derived form, those same enhancers instead interact with AGAP007066, and ED1 additionally interacts with AGAP007064 and AGAP007063 (Fig2B). Enhancer-promoter interactions occurring over 2La inversion breakpoints were confirmed using an independent computational method, PSYCHIC^69^. Specifically, AGAP005781 is predicted to interact with an enhancer across the distal breakpoint in 2La fixed cells and across the proximal breakpoint in 2L+^a^ fixed cells, and AGAP007066 interacts with an enhancer across the proximal breakpoint in 2La fixed cells. For each of these enhancers with evidence of reciprocal rewiring associated with the inversion, we measured enhancer activity by luciferase assay, comparing the activities of the 2L+^a^ and 2La alleles in a 2L+^a^/2L+^a^ cellular background and a 2La/2La cellular background by one-way ANOVA followed by Tukey’s multiple comparison test (Fig2C). We find examples of enhancers with differential activity regardless of cell line assayed in (e.g. EP1, EP2, and ED1), an enhancer with the same activity regardless of cell line assayed in (e.g. ED3), and an enhancer with activity differences when assayed in 2La cells but not in 2L+^a^ cells (e.g. ED2). The most biologically relevant cases are the innermost bars in which enhancer alleles are tested in their respective cell lines. When comparing the 2L+^a^ and 2La alleles assayed only in their respective cell lines, the EP1 2L+^a^ allele has significantly higher activity, EP2 and ED1 2La alleles display significantly higher activity, and the enhancer activities of ED2 and ED3 are not significantly different between 2L+^a^ and 2La alleles. Given the ectopic nature of a luciferase assay, conclusions regarding the effect of an enhancer on target gene expression cannot be extrapolated as these would need to consider aspects of chromatin accessibility. While additional work is needed to validate the functional interaction between candidate enhancers and their target genes, we have performed ATAC-seq on these cell lines and have shown that the 5 candidate enhancers and 4 promoter regions are all in regions of open chromatin, further supporting these enhancer-promoter interactions as being active. All five candidate enhancers overlap with regions of open chromatin supported by our 2La and 2L+^a^ cell line ATAC-seq peaks (Fig2B). Furthermore, each candidate enhancer is also independently supported by one or more of previously published ATAC-seq peaks (ours^50^ and others^17^), FAIRE-seq peaks^51^, and H3K27ac ChIP-seq peaks^16^ from other labs, suggesting they are active enhancers across multiple tissues and cell types. Analyzing enhancer and promoter regions for overlap with differential ATAC-seq peaks, only EP1 and AGAP005781 overlap with differential ATAC-seq peaks, both of which are more accessible in 2L+^a^ cell line samples as compared to 2La cell line samples, which is consistent with the significantly higher activity of EP1 in 2L+^a^ cells and significant upregulation of AGAP005781 in 2L+^a^ cells (Fig2C). The current measures of differential accessibility are a snapshot, assessing these two cell lines (Ag55 and Sua4.0) under basally growing conditions; sampling following immune stimulation could lead to different/additional differentially accessible regions. All 2La and 2L+^a^ cell line ATAC-seq peaks and differential ATAC-seq peaks can be found in Additional File 3, Table S4. The observed phenomenon that all 5 enhancers (EP1, EP2, ED1, ED2, and ED3) involved in enhancer reciprocal rewiring lie outside of the inversion is related to the distribution of STARR-seq enhancers inside and outside the 2La inversion breakpoints. Enhancers inside the inversion are located further from the breakpoint (nearest enhancer is ~31kb from the proximal breakpoint and ~254kb from the distal breakpoint) compared to enhancers external to the inversion (nearest enhancer is ~4kb from the proximal breakpoint and ~3kb from the distal breakpoint). The farther distances to a breakpoint coupled with the criteria requiring reciprocal rewiring, limited the inclusion of enhancers located within the 2La inversion. Enhancers within the 2La inversion are involved in non-reciprocal rewiring, which is explored in the next section. A complete list of raw interaction counts for the 13 interactions involved in enhancer reciprocal rewiring, with their log_2_FoldChange and adjusted p-values, is in Additional File 3, Table S5. Interactions are identified from the Micro-C contact matrices, and as demonstration of this, an enlarged image of the contact matrix indicating interaction between AGAP005781 and ED1/ED2/ED3 in Sua4.0 (2La/2La) samples mapped to PEST is presented in Additional File 1, FigS4.

To explore whether TAD structure changed as a result of the 2La chromosomal inversion, we compared TADs from 2L+^a^ fixed samples mapped to PEST (2L+^a^ reference genome) with TADs from 2La fixed samples mapped to MOPTI (2La reference genome) using HiCExplorer hicFindTADs^70–72^ as the TAD calling algorithm. Analysis was performed on samples mapped to the reference genome matching their 2La inversion karyotype so that read mapping would not interfere with TAD calling. While there is no evidence of a TAD being newly created nor destroyed (i.e. TADs span the proximal and distal breakpoints in both 2La and 2L+^a^ cell line samples), TAD structure around the 2La inversion breakpoints significantly changed. For the proximal breakpoint, 53.2% of the breakpoint-spanning TAD (60kb total size) is within the 2La inversion in 2La samples while in 2L+^a^ samples, 65.6% of the breakpoint-spanning TAD (60kb total size) is within the inversion (p<0.0001) (Additional File 1, FigS5). For the distal breakpoint, in 2La samples, 0.5% of the breakpoint-spanning TAD (115kb total size) is within the 2La inversion while in 2L+^a^ samples, 2.0% of the breakpoint-spanning TAD (110kb total size) is within the inversion (p<0.0001) (Additional File 1, FigS5). Perhaps more biologically relevant are the difference in TAD composition between 2La and 2L+^a^ breakpoint-spanning TADs. For 2L+^a^ samples mapped to PEST, in the proximal breakpoint-spanning TAD, there are 4 promoter regions and 1 STARR-seq identified enhancer within the inversion (marked by asterisks in Additional File 1, FigS5) which, due to the chromosomal inversion in 2La samples are at the distal end of the inversion and therefore no longer in the proximal breakpoint-spanning TAD. In the distal-breakpoint containing TAD in 2L+^a^ samples, there is 1 promoter region within the inversion (marked by asterisks in Additional File 1, FigS5) which in 2La samples lies at the proximal end of the inversion and therefore no longer in the distal breakpoint-spanning TAD. Related to the current work is the conclusion that the identified 13 differential interactions involved in enhancer reciprocal rewiring over 2La breakpoints include interactions that occur between TADs (Additional File 1, FigS6). Specifically, in 2L+^a^ samples, EP1 and AGAP005781 interact over a TAD boundary, and the interactions between ED1/ED2/ED3 and AGAP007066/AGAP007064/AGAP007063 occur over TAD boundaries. In 2La samples, EP1 interacts with AGAP007066, AGAP007064, and AGAP007063 over a TAD boundary and ED1, ED2, and ED3 interact with AGAP005781 over a TAD boundary (Additional File 1, FigS6). A/B compartment analysis, which identifies active and inactive chromatin, respectively, confirmed that all 13 interactions occur within A compartments, as expected (Additional File 1, FigS6).

### Broader exploration into novel enhancer rewiring associated with chromosome 2 inversions

Strict filtering criteria were implemented in the above investigation of enhancer rewiring occurring over 2La inversion breakpoints to distill the 329 significant differential interactions over the breakpoint into manageable, tractable interactions. Now that enhancer rewiring over inversion breakpoints has been demonstrated, we relaxed the filtering criteria to more generally explore the following 3 scenarios: (1) enhancer rewiring involving genes not differentially expressed between cell line samples by RNA-seq, (2) enhancer-promoter interactions spanning inversion breakpoints detected in one cell line but not the other, and (3) enhancer rewiring over chromosomal inversion 2Rbc. Relaxing the criteria to also include interactions involving nonDEG promoters but still limiting criteria to instances of enhancer reciprocal rewiring over 2La inversion breakpoints identified 7 additional interactions. Notably, the same 5 enhancers involved in enhancer reciprocal rewiring with DEGs were also involved in enhancer reciprocal rewiring with nonDEGs (Table 1). While these genes were not differentially expressed between 2La and 2L+^a^ samples under the conditions tested, they may be differentially expressed in other cell types or conditions. The additional nonDEGs in these interactions are AGAP029526, AGAP007068, AGAP007065, and AGAP007062, which are each generally related to the regulation of cuticle remodeling. AGAP029526 is an unspecified product. AGAP007068 is RING finger protein 113A. AGAP007065 is transcription factor CP2 and related proteins. AGAP007062 is ecdysis-triggering hormone (ETH). Cuticle remodeling is an important process for maintaining the physical barrier against desiccation, so the enhancer rewiring involving these genes may be a possible mechanistic explanation for why the 2La inversion has been associated with aridity and thermal tolerance^18, 28^. The gene name, log_2_FC, and p.adj for these nonDEGs involved in reciprocal enhancer rewiring can be found in Additional File 3, Table S6.

Of the 329 significant differential interactions over a 2La inversion breakpoint, 48 interactions were valid enhancer-promoter pairs (i.e. one anchor contained an enhancer while its partner anchor contained a promoter), 41 were asymmetric interactions (i.e. one anchor contained both an enhancer and a promoter while its partner anchor did not contain an annotated promoter nor a STARR-seq enhancer), 174 were single element interactions (i.e. one anchor contained either an enhancer or a promoter while its partner anchor did not contain an annotated promoter nor a STARR-seq enhancer), and 66 were interactions lacking annotation of genes and regulatory elements (stacked bar graph, Additional File 1, FigS7; table of interactions with logFC and p.adj, Additional File 3, Table S7). It is important to note that while some interactions/anchors appear to lack annotated features underlying the functional interaction between chromosome regions, this could be the result of a number of factors including the field’s currently limited annotation of known regulatory elements, a mis-annotation of the reference genome, and/or poor annotation of lncRNA genes and other non-protein coding elements.

Given that the 2Rbc region of the 2R chromosome arm was also inverted in the examined samples, enhancer rewiring was explored in this inversion with distinct evolutionary history^36^. Of the 192 significant differential interactions over a 2Rbc breakpoint, 10 interactions were valid enhancer-promoter pairs, 2 were asymmetric interactions, 81 were single element interactions, and 99 were interactions lacking annotation of genes and regulatory elements (stacked bar graph, Additional File 1, FigS7; table of interactions with logFC and p.adj, Additional File 3, Table S8). The distribution of differential interaction categories is significantly different between chromatin interactions occurring over the 2Rbc breakpoint compared to over the 2La breakpoint (p<0.0001), where the 2Rbc inversion analysis shows a greater number of differential interactions lacking annotation than does the 2La analysis. Valid enhancer-promoter pairs occurring over 2La or 2Rbc breakpoints were then further analyzed to identify enhancers involved in rewiring to identify enhancer-promoter interactions either fully disrupted or newly created by the inversion (Tables 2&3). Genes involved in enhancer rewiring over 2La breakpoints have broad functions including metabolism and biosynthesis, lysosomal degradation, cell signaling and circadian rhythm, ion transport and homeostasis, apoptosis, and gene regulation. Of note, AGAP007059 interacts with ED1 over the distal breakpoint in 2L+^a^ samples but does not interact with any enhancers over the breakpoint in 2La samples. We have shown previously that AGAP007059 activity is antagonistic to Plasmodium infection^73^. As for the 2La inversion, the genes involved in enhancer rewiring over 2Rbc breakpoints display broad functions, including enzymatic activity regulation, receptors and membrane transport, and cellular structure. Gene functions can be found in Additional File 3, Table S3.

### Applying differential interaction analysis to differentially expressed LRR genes within the 2La inversion with characterized *Plasmodium* function

While the genes involved in differential enhancer-promoter interactions spanning the 2La inversion breakpoints have broad functions that may directly or indirectly influence one or more of the intrinsic or extrinsic vector competence factors associated with the 2La inversion, we also took a complementary approach centered on known leucine-rich repeat (LRR) immune genes within the 2La inversion that have been shown to be *Plasmodium* antagonists, specifically APL1A (AGAP007036)^3–8^, APL1B (AGAP007035)^3–8^, APL1C (AGAP007033)^3–8^, and LRIM1 (AGAP006348)^3–6, 10, 12^. Bulk-RNA seq analysis showed that APL1A, APL1B, and LRIM1 were all significantly upregulated in 2La cells (|log2FC|>1, p<0.001) while APL1C was downregulated in 2La cells, though not significantly so according to a log_2_FC cut-off of 1 (|log2FC|=0.67, p=0.04). All four genes were involved in differential chromatin interactions across inversion karyotypes, all of which were more frequent in 2L+^a^ cells except for one differential interaction from the LRIM1 promoter that was more frequent in 2La cells (Additional File 3, Table S9). Due to the fact that all interactions contained a promoter region, we next assessed whether the interactions were valid enhancer-promoter pairs, single-element interactions, or asymmetric interactions. Of the four differential APL1A interactions, two were valid enhancer-promoter pairs while two were single-element interactions lacking an enhancer. Of the fifteen differential APL1B interactions, one was a valid enhancer-promoter pair while fourteen were single-element interactions lacking an enhancer. Of the eight differential APL1C interactions, two were valid enhancer-promoter pairs while six were single-element interactions lacking an enhancer. Of the three differential LRIM1 interactions, one was an asymmetric interaction and two were valid enhancer-promoter pairs (Additional File 3, Table S9). However, one of the two valid enhancer-promoter pairs was an interaction between an enhancer found in the LRIM1 promoter anchor and four genes (AGAP006353, AGAP006354, AGAP006355, AGAP006356) found in the partner anchor. Because there was no STARR-seq enhancer in the partner anchor to the LRIM1 promoter, this interaction was not considered for further analysis.

In total, the six valid enhancer-promoter pairs for the LRR genes involved six enhancers, all of which overlap with our 2La and 2L+^a^ cell lines ATAC-seq peaks. The six candidate enhancers additionally overlap with at least one of the following previously published chromatin accessibility datasets: ATAC-seq peaks (ours^50^ and others^17^), FAIRE-seq peaks^51^, and H3K27ac ChIP-seq peaks^16^ from other labs. However, none of the ATAC-seq peaks displayed significantly different accessibility between 2La and 2L+^a^ cell line samples, suggesting that the differential gene expression between 2La and 2L+^a^ samples is more likely due to enhancer allele differences in activity and/or differences in transcription factor availability rather than a differential accessibility to the enhancers. Additionally, these differential interactions occur well within the bounds of the 2La inversion (LRIM1 is ~9.8MB to the nearest breakpoint and the APL1 gene family is ~892kb to the nearest breakpoint), making allelic differences between inversion karyotypes a reasonable explanation for the differential interactions; however, cascading effects of chromatin conformation changes cannot be excluded.

### Exploration of differential enhancer-promoter pairs with APL1A, APL1B, APL1C, and LRIM1 using distance-decay correction-analysis on Micro-C interaction frequencies

To determine whether the differential enhancer-promoter interactions involving known *P. falciparum* antagonists were enriched compared to the null distribution of all interaction frequencies from each promoter region, we performed a novel distance-decay correction analysis on the Micro-C and determined interaction frequencies for all interactions anchored on a given LRR promoter region (APL1A, APL1B, APL1C, and LRIM1 as above). Because chromatin interactions decay with increasing physical distance, as proximal regions inherently interact more frequently, raw Hi-C or Micro-C contact matrix values must be distance-normalized to accurately identify interactions with elevated interaction frequencies. Briefly, the distance-decay correction analysis identifies chromatin interactions that are enriched (defined as occurring more frequently than expected for the distance between the two interacting regions) using linear regression analysis on log-transformations of interaction frequencies and their distances to establish a local null distribution for each anchor region’s interactions, against which the observed interaction frequencies are compared. Resulting z-scores, which describe, in standard deviation units, how frequent a given interaction is, define enrichment where a z-score>0 indicates an interaction occurred more frequently than expected for its distance and a z-score>1.96 indicates a statistically significant enrichment (p<0.05). Further details on this distance decay method are outlined in Methods. Importantly, we do not expect all biologically relevant interactions to reach statistical significance because enrichment is determined from the local null distribution of interaction frequencies from the given anchor. As one enhancer can regulate multiple genes and a single gene can be regulated by multiple enhancers, there may be multiple biologically relevant high-frequency interactions, thus inflating the variance of the background null distribution and preventing those high-frequency interactions from exceeding the traditional two-standard-deviation threshold for significance.

The two APL1A differential interactions containing an enhancer were mildly enriched, as defined in Methods (interaction with partner anchor 2L: 41230000- 41235000, z-score=0.62, p=0.53; interaction with partner anchor 2L: 41330000-41335000, z-score = 0.52, p=0.61). The single APL1B differential interaction containing an enhancer occurred less frequently than expected for its distance (interaction with partner anchor 2L: 41225000-41230000, robust z-score=−0.25, p=0.80). One of the two APL1C differential interactions containing an enhancer was mildly enriched (interaction with partner anchor 2L: 41330000-41335000, z-score=0.32, p=0.75) while the other was mildly underrepresented (interaction with partner anchor 2L: 41225000-41230000, z-score= −0.71, p=0.48). The one LRIM1 differential interaction containing an enhancer was mildly enriched (interaction with partner anchor 2L: 30470000- 30475000, z-score=0.68, p=0.50) (Additional File 3, Table S10). The lack of significant z-scores indicates that that are more frequent interactions that are not differential.

### Distance decay correction analysis identifies candidate enhancers for *Plasmodium* antagonists

To better understand the regulation of LRIM1, we broadened our exploration of chromatin interactions by analyzing all interactions, not only the differential interactions considered above, and identify the top candidate enhancer. We identified the partner anchors that contained a STARR-seq identified enhancer and ranked interactions by z-score to identify the top candidate enhancer defined as the enhancer found in the most enriched interaction. Using this approach *in lieu* of the simpler nearest gene model can pinpoint physical evidence-based candidate regulatory elements worthy of further investigation. When anchoring on the 1000bp region upstream of LRIM1 as a proxy for its promoter, the 5kb enhancer-containing region with the highest z-score contains an enhancer with coordinates 2L: 30142958-30143475 (z-score 2.16, p-value 0.03), located 187,821bp downstream of the LRIM1 gene (Fig3A). Additionally, this candidate enhancer overlaps with a 2La and 2L+^a^ cell line ATAC-seq peak, though not a differential ATAC-seq peak, as well as with previously published ATAC peaks^17, 50^, FAIRE-seq peaks^51^, and H3K27ac ChIP-seq peaks^16^, suggesting that it is an active enhancer (Fig3A). Activities of both 2La and 2L+^a^ alleles of this candidate LRIM1 enhancer were confirmed by luciferase assays conducted in both 2La/2La and 2L+^a^/2L+^a^ cell lines. The 2La allele assayed in 2L+^a^ cells had significantly lower activity compared to the other allele and cell line combinations. The most biologically relevant comparison, however, is between alleles assayed within their respective cell lines, which are not significantly different (Fig3B). Reciprocally, when interactions were instead anchored on the newly identified candidate LRIM1 enhancer, the 5kb region containing LRIM1’s promoter displayed a high interaction frequency for its distance (robust z-score 2.24, p=0.02), which is the 2^nd^ highest z-score of 5kb regions containing a promoter (Fig3C). The 5kb promoter-containing region with the highest z-score contains the promoter for AGAP006346, the homologue of *D. melanogaster* spinster (robust z-score 3.38, p = 0.0007). Multiple high frequency interactions from a regulatory region are expected as it is not uncommon for an enhancer to participate in regulating transcription of multiple target genes^49, 74^. This observed reciprocity of enriched interaction further supports 2L: 30142958-30143475 as a candidate enhancer of LRIM1. Additional functional validation work is needed to confirm LRIM1 as a target gene of this candidate enhancer. However, here we demonstrate the novel utilization of proximity ligation sequencing data to identify and prioritize candidate enhancers not otherwise considered previously.

The top candidate enhancers for the 3 member APL1 gene family, APL1A, AP1B, and APL1C, were identified in the same manner as for LRIM1. Given that the 3 member APL1 gene family arose via gene duplication within the A. gambiae species complex^7^,unsurprisingly APL1A, B, and C share a top candidate enhancer (2L: 41279774-41280274; APL1A z-score=1.65, p=0.10; APL1B robust z-score=1.49, p=0.14; APL1C z-score=1.08, p=0.28) (Fig4) located 5,873bp upstream of APL1A, 10,410bp upstream of APL1B, and 18,589bp upstream of APL1C. For the APL1 gene family, the top candidate enhancer is the nearest enhancer; however, that is not always the case as was demonstrated by analysis of LRIM1. The candidate APL1 common enhancer overlaps with a 2La and 2L+^a^ cell line ATAC-seq peak as well as previously published FAIRE-seq peaks^51^ and H3K27ac ChIP-seq peaks^16^, suggesting that it is an active enhancer. This enhancer overlaps a differential ATAC-seq peak that is more open in 2L+^a^ cell line samples (Fig4B). The promoters of APL1A, APL1B, APL1C also overlap with differential peaks more open in 2L+^a^ samples (Fig4B). While it remains unclear how this differential accessibility relates to differential gene expression, the presence of these enhancer-promoter interactions in regions of open chromatin further supports these as active enhancer-promoter interactions. Measuring enhancer activity of both 2La and 2L+^a^ alleles of the candidate APL1 common enhancer in both 2La and 2L+^a^ cellular backgrounds by luciferase assay, the 2La allele displays significantly higher activity than the 2L+^a^ allele when assayed in their respective cellular backgrounds, the most biologically relevant comparisons. The difference in activity is at least partially due to sequence differences between 2La and 2L+^a^ alleles. Allelic differences may be cell line specific and/or associated with 2La inversion karyotype.

### Newly identified candidate LRIM1 enhancer contains conserved sequence related to immune regulation

Despite its large physical distance from LRIM1 (AGAP006348, PEST positions 30329656-30331296), the newly discovered candidate enhancer (2L: 30142958-30143475) contains conserved sequence regions related to immune regulation. A ~90bp region (PEST positions 30143151-30143240) has high levels of sequence conservation (e values < 4×10^−4^) amongst members of the *A. gambiae* complex, two species most closely related to the *A. gambiae* complex (*A. christyi and A. epiroticus*), and more distantly related anopheline vectors of malaria (*A. stephensi*^75^ and *A. funestus*^76^). Given that regulation of LRIM1 via both the Rel1 and Rel2 pathways has been described^77, 78^, we examined this conserved sequence region for evidence of Rel1 (*Drosophila* Dif) or Rel2 (*Drosophila* Relish) TFBSs using MEME Suite^79^ and available data from *Drosophila melanogaster* in JASPAR^80^. Within this conserved region, 6 of the 8 species share significant sequence similarity (p<0.01) with a TFBS for the Relish (*Anopheles* Rel2) transcription factor (MA2264.1) at the 5’ end of the sequence, and 5 of the 8 species show significant similarity with the Relish TFBS on the 3’ end (p<0.01) (Fig5). A query of the other NF-kB-like TFBS (MA2213.1) in *Drosophila*, Dif (*Anopheles* Rel1), shows 7 of the 8 species share significant similarity (p<0.01) only at the 5’ end (Fig5). For all members of the *A. gambiae* species complex as well as *A. christyi* and *A. epiroticus*, both Dif and Relish binding sites are significant while for *A funestus,* only Dif is significant, and for *A. stephensi*, only Relish is significant (Fig5).

The ~180kb distance between the LRIM1 gene and the Micro-C-identified LRIM1 candidate enhancer is also conserved across 6 of the *Anopheles* species tested, minimally 173,257bp in *A. coluzzii* MOPTI to maximally 186,416bp in *A. gambiae* PEST. It is impossible to determine the distance between LRIM1 and this candidate regulatory region in either *A. christyi* or *A. epiroticus* given that the sequences are present on distinct sequence contigs.

Performing the same analysis on the candidate APL1 common enhancer, we found high conservation of the entire APL1 common enhancer fragment within the *Anopheles gambiae* species complex (e<9e-119). However, there are no fragments >50bp that are conserved in *A. stephensi* or *A funestus,* two distantly related anopheline malaria vectors outside of the species complex. The differing levels of conservation may be the result of the evolution of the APL1 gene family, which originated as a single APL1 gene and duplicated within the *Anopheles gambiae* species complex, giving rise to the 3 genes APL1A, APL1B, and APL1C^7^.

### Target gene identification of 9 previously characterized enhancers within the malaria susceptibility locus on chromosome arm 2L

Capitalizing on the novel distance-decay correction analysis method, we identified the top candidate target genes of the 9 previously characterized enhancers within the malaria susceptibility locus on chromosome arm 2L.^50, 81^ Top candidate target genes were identified by the highest z-score of promoter-containing interactions when anchored on each enhancer. Among these genes are AGAP005749 which is glutathione S-transferase omega class 1, AGAP007050 which is a glucosyl/glucuronosyl transferase, AGAP007040 which is cuticular protein RR-1 family 61, AGAP007058 which is homeobox protein DLX, and AGAP007032 which is a breast cancer 2 susceptibility protein. These genes are generally related to metabolic detoxification^82–84^, response to environmental stress^85–87^, and tissue remodeling^88^. The top candidate target gene(s), their associated z-score, and their approximate distance from the enhancer are listed in Table 4. Because analysis is performed at 5kb resolution, the nearest possible candidate target gene identified in this analysis would be in the adjacent 5kb region to the enhancer. The distances of candidate target genes from their enhancer ranged from ~4-564kb, demonstrating the variety of distances from which enhancers can act on their target gene(s)^89, 90^.

## DISCUSSION

The 2La chromosomal inversion is a large, monophyletic, polymorphic inversion segregating in both *A. coluzzii* and *A. gambiae* mosquitoes, the two major malaria vectors in Sub-Saharan Africa where malaria morbidity and mortality are highest. This paracentric inversion is associated with an intrinsic resistance to malaria infection^25, 26^, among other important phenotypes contributing to the mosquito’s ability to transmit malaria, including feeding and resting behavior, insecticide resistance and temperature and aridity tolerance^18, 25, 27–31^. How the 2La inversion alters enhancer-mediated gene regulation remains unknown.

While chromatin conformation capture techniques have been used to study fine-scale chromatin interactions, such as enhancer-promoter interactions, in other organisms^46–48^, to date, these proximity ligation techniques have only been used in *Anopheles* mosquitoes for broader scale applications like chromosome assemblies^41–44^, inversion breakpoint mapping^36^, determining TAD structure^42^, and broad-scale analysis of chromatin loops^42^. Our fine-scale exploration of chromatin interactions within *A. coluzzii* hemocyte-like cells complements more broad-scale applications by identifying enhancer-promoter interactions involved in reciprocal rewiring over 2La inversion breakpoints as well as utilizing proximity-ligation data and analytical methods to probe enhancer-promoter interactions for known *Plasmodium* antagonist genes within the 2La inversion and in regions of the 2L inversion previously associated with malaria susceptibility in wild mosquitoes.

### Chromatin interaction and transcriptional differences over chromosome 2 inversions 2La and 2Rbc

The multifaceted approaches used here identified significant enrichment of differential chromatin interactions over the monophyletic 2La inversion but no enrichment of differentially expressed genes. This is likely due to the physical rearrangement of the 2La chromosomal inversion having a direct effect on chromatin interactions while changes in gene expression being the sum of a cascade of direct and indirect effects on gene expression^91^. For the younger 2Rbc chromosomal inversion with evidence of breakpoint reuse^36^, neither differential interactions nor differentially expressed genes were enriched. The lack of enrichment over the 2Rbc inversion compared to the significant enrichment over the 2La inversion likely results from the differing evolutionary history of the two inversions. The 2La inversion, based on breakpoint sequence analysis has been reported to have a monophyletic origin^22, 25^, resulting in divergence of inverted and non-inverted chromosomes, while 2R inversions have been reported to involve breakpoint re-use^36^, allowing for some degree of homogenization as inversions can revert to their ancestral orientation as was the case for 2R+^b32^. It should be noted that while Ag55 (harboring karyotypes 2L+^a^/2L+^a^ and 2Rbc/2Rbc) and Sua4.0 (karyotypes 2La/2La and 2R+^bc^/2R+^bc^) hemocyte-like cell line samples are instances of the 2La and 2Rbc inversion karyotypes, they may not be representative of all 2La or 2Rbc inversion karyotypes. Therefore, some observed differences may be cell line-specific and not representative of 2La or 2Rbc inversion karyotype differences in general.

### Enhancer rewiring and the 2La and 2Rbc chromosomal inversions

The function of enhancers to modulate gene expression enables fine tuning of gene transcription, offering a level of adaptability to the organism. Additionally, the alteration of an enhancer’s target gene(s) through rewiring allows for plasticity of the population on an evolutionary scale^92^. In the context of cancer, a sort of micro-evolution occurring within an organism, it has been demonstrated that enhancer rewiring is a likely factor contributing to cancer progression and metastasis^93^. Large clusters of enhancers controlling cell lineages (called super-enhancers), have been shown to be key drivers of gene expression changes that sustain the persistent survival of cancer cells^94^. Chromosomal inversions and the differential phenotypes associated with them have been found to play an important role in adaptation^95–97^. Enhancer rewiring occurring at inversion breakpoints is likely one of many mechanisms, among which include the evolutionary divergence of inversion-captured genetic variation and co-adapted gene complexes, by which chromosomal inversions offer fitness and evolutionary advantages^20, 21, 98, 99^.

Here, we demonstrate reciprocal rewiring of a set of enhancers and genes across 2La inversion breakpoints. There are multiple non-mutually exclusive hypotheses that could explain the disruption and creation of these inversion karyotype-specific enhancer-promoter interactions. The differential expression of target genes could be the result of distinct enhancers regulating genes across alternate inversion karyotypes. The inversion could have been selected for fitness advantages unrelated to these 4 target genes, but it is also possible that these 4 target genes are essential to mosquitoes that regulation of them needed to be maintained, thereby necessitating enhancer re-wiring to ensure gene expression. While enhancer function is thought to be orientation independent (sense vs antisense)^74, 100^, investigation of human and *Drosophila* enhancers shows that enhancer positioning (upstream vs downstream) can impact transcriptional kinetics and gene expression, which may be context specific^100–103^. Notably, reduced RNA transcription has been observed in enhancers downstream from genes^101, 102^. For three of the four genes involved in reciprocal rewiring (AGAP005781, AGAP007066, AGAP007063), this enhancer rewiring re-establishes an interaction between each promoter and an upstream enhancer, thereby potentially ensuring stable gene expression. It has also been shown that genomes utilize enhancer redundancy or “shadow enhancers” to safeguard gene expression against genetic or environmental disruptions^104–107^. If a genetic or environmental change disrupts the interaction between a target gene and its primary enhancer, the gene expression would normally be altered. However, because of shadow enhancers, potentially established by the observed enhancer rewiring here, the target genes can maintain stable expression even in cases of stress. We also demonstrate instances of enhancer-promoter interactions over 2La breakpoints that are either abolished or newly created, highlighting the complexity and plasticity of enhancer-promoter interactions^108^. In the context of the 2Rbc chromosomal inversion, we do not find evidence of enhancer- promoter reciprocal re-wiring, likely due to breakpoint re-use resulting in the lack of a single origin as described above. However, we do find instances of newly created or abolished enhancer-promoter interactions of 2Rbc breakpoints, suggesting that chromosomal inversion events are not restricted to regions lacking regulatory element interaction and that enhancer-promoter interactions are dynamic^108–110^.

### Utilizing interaction frequencies to prioritize candidate regulatory regions

Despite the previous identification and functional characterization of a number of *A. gambiae* genes on the 2L chromosome arm with *Plasmodium* antagonist function^4–8, 10–13^, comparatively little is understood about how these genes are regulated or what their candidate regulatory elements may be. Consistent with the nearest enhancer model, our newly identified candidate APL1 common enhancer is the nearest STARR-seq enhancer to APL1A, APL1B, and APL1C. However, this is not the case for our newly identified LRIM1 candidate enhancer where there are 7 enhancers closer to the LRIM1 promoter region than our newly identified candidate enhancer. Previous studies have queried the most proximal enhancer (2L: 30333431-30334787), located 1,135bp upstream of the LRIM1 promoter region, as LRIM1’s candidate enhancer^17, 111^. However, application of a novel distance-normalized interaction frequency approach facilitated discovery of an LRIM1 candidate enhancer, located ~188kb downstream from the promoter. Such a distance is not unreasonable for an enhancer-promoter interaction because others have reported long-range interactions of >100kb in *Drosophila*^112, 113^, and *A. coluzzii* TADs sizes have been reported to be up to 1MB in size^42^. This novel interaction frequency-based approach prioritizes a candidate LRIM1 enhancer over 7 STARR-seq identified candidate enhancers that are closer to the LRIM1 promoter region along the linear chromosome (5 upstream and 2 downstream) than the candidate regulatory region identified here (Fig3A).

While cementing the relative importance of the newly discovered LRIM1 regulatory element and the shared APL1 candidate enhancer will require additional work, it is only through proximity ligation assays like the Micro-C performed here that we are able to prioritize regulatory elements at a distance from the genes they regulate and prioritize enhancer-promoter interactions for future investigation.

### Conclusions

Here, we utilize high-resolution measures of chromatin conformation alongside gene expression to characterize the impact of chromatin conformational changes associated with the 2La and 2Rbc inversions on gene expression and 3D chromatin structure. Coupling chromatin conformation and gene expression results from 2La and 2L+^a^ *A. coluzzii* hemocyte-like cell lines in the case of the monophyletic 2La inversion, we identify sets of genes and enhancers that differentially interact over inversion breakpoints, suggestive of enhancer rewiring and the novel evolution of enhancer-promoter interactions over inversion breakpoints. The lack of such reciprocal rewiring with the 2Rbc inversion may reflect that unique evolutionary histories of chromosomal inversions have differing effects on enhancer re-wiring while also highlighting the plasticity of enhancer-promoter interactions in the context of chromosomal inversions. Further investigation into these enhancers/genes, or other genomic elements near inversion breakpoints, could help determine whether enhancer re-wiring in *A. coluzzii* mosquitoes produces stable inversion-independent gene expression changes or instead establishes an inversion-specific regulatory program driven by modified target gene relationships. Further capitalizing on novel proximity ligation sequencing data, we demonstrate how interaction frequencies can be utilized to prioritize candidate enhancers/genes for the LRIM1 gene, the APL1 gene family, and 9 previously characterized enhancers within the malaria susceptibility locus, all of which are on chromosome arm 2L. These novel approaches allow fine-scale exploration of mosquito chromatin interactions, providing insight into the evolution of enhancer-promoter interactions as well as the unique regulatory circuitry across inversion forms, both of which aid in the design of evolutionarily informed vector control tools potentially targeting mosquitoes of a given karyotype. Furthermore, these novel analyses of proximity ligation sequencing are broadly applicable across species for which Micro-C/Hi-C data are available.

## METHODS

### Cell culture

Two hemocyte-like *A. coluzzii* cell lines Sua4.0 (cell line homozygous for the 2La karyotype, will be referred to as 2La cells throughout)^53, 54^ and Ag55 (cell line homozygous for the 2L+^a^ karyotype, will be referred to as 2L+^a^ cells throughout)^54–56^ were seeded at a density of 0.9×10^6 cells/mL in a T75 flask (three flasks per cell line per replicate) in antibiotic-free media (Insect Xpress (Lonza) for Sua4.0, and Leibovitz’s L-15 Medium (Millipore Sigma) for Ag55). 48hr post-seeding, cells were washed with 1X PBS, scraped, and aliquots of ~1million cells were spun at 3,000g for 5min.

Supernatants were removed and cell pellets flash frozen in liquid nitrogen and stored at −80C until shipment for bulk RNA-sequencing (Azenta, South Plainfield, NJ) or Micro-C (Dovetail Genomics, part of Cantata Bio, Scotts Valley, CA). Four biological replicates were submitted for RNA-seq with the three most similar RNA-seq replicates (as determined by PCA analysis, Additional File 1, FigS8) also submitted for Micro-C. For ATAC-seq, cells were seeded at the same density as for RNA-seq and Micro-C (0.9×10^6 cells/mL), but instead of flash freezing, cells (ranging from 3.7-6.8×10^6 cells) were frozen in cryopreservation solution (50% FBS, 40% media, 10% DMSO) and stored at −80C until shipment for bulk ATAC-seq (ActiveMotif, Carlsbad, CA). Two biological replicates for each cell line were submitted. Importantly, to our knowledge there are no 2La polymorphic cell lines and no mosquito colonies segregating for the 2La inversion available in public repositories.

### RNA-seq

#### RNA-sequencing library preparation and sequencing

Azenta performed RNA extraction, strand-specific library preparation, and sequencing to a depth of ~20 million paired-end reads per sample from flash frozen cell pellets of either Sua4.0 cells (2La) or Ag55 cells (2L+^a^). Four biological replicates were performed for each cell line. Briefly, total RNA was extracted, and mRNA was enriched by Poly(A) selection. RNA integrity was assessed via TapeStation, and all samples had an RIN ≥ 9. After library preparation, sequencing was performed on an Illumina NovaSeq platform to generate 2×150bp paired end reads. 17.7–18.9million reads were obtained per sample.

#### Read preprocessing alignment and quantification

Raw reads were assessed for quality using FastQC^114^. All samples showed high per-base quality (≥90% bases above Q30), and all reads had a mean quality score >35. Adapter sequences and low-quality bases were trimmed using Trimmomatic-0.38^115^ with parameters ILLUMINACLIP:TruSeq3-PE.fa:3:30:10:2:keepBothReads LEADING:3 TRAILING:3 MINLEN:26. Post-trimming quality was reassessed using FastQC. Cleaned reads were aligned to PEST version AgamP4 (VectorBase release 68)^67, 68^ using STAR (v2.7.10b)^116^, resulting in 90.26%−92.18% uniquely mapped reads. Annotation was based on VectorBase-68_AgambiaePEST.gff. Gene-level read counts were obtained using featureCounts (v2.0.6)^117^.

#### Differential expression analysis

Raw counts data were loaded into R, then normalized and analyzed using DESeq2 (v1.46.0)^63^. Prefiltering removed genes with fewer than 10 reads total, summed across all 8 samples being compared. DEGs between hemocyte-like cells homozygous for the 2La karyotype and those homozygous for 2L+^a^ were defined as having a |log_2_foldchange| ≥1 and adjusted p-value ≤0.05. Plotting the genomic position of significantly up- and down-regulated genes was performed using R packages tidyverse (v2.0.0)^118^ and ggrepel (v0.9.6)^119^. All RNA-seq reads are accessible at PRJNA1439337.

### Micro-C

#### Micro-C library preparation and sequencing

Because 2–3 biological replicates is standard for Hi-C/Micro-C, we performed PCA analysis on our four RNA-seq samples to determine the three most similar biological replicates (Additional File 1, FigS8). Biological replicates 1, 2, and 3 were submitted to Dovetail Genomics for Micro-C library preparation and sequencing. Briefly, the chromatin was fixed with disuccinimidyl glutarate (DSG) and formaldehyde in the nucleus. The cross-linked chromatin was then digested in situ with micrococcal nuclease (MNase). Following digestion, the cells were lysed with SDS to extract the chromatin fragments, and the chromatin fragments were bound to Chromatin Capture Beads. Next, the chromatin ends were repaired and ligated to a biotinylated bridge adapter followed by proximity ligation of adapter-containing ends. After proximity ligation, the crosslinks were reversed, the associated proteins were degraded, and the DNA was purified then converted into a sequencing library using Illumina-compatible adaptors. Biotin-containing fragments were isolated using streptavidin beads prior to PCR amplification. The library was sequenced on an Illumina NovaSeq platform to generate 201–234 million 2 × 150bp read pairs per sample. Samples were 3 biological replicates each of hemocyte-like cells homozygous for the 2La karyotype and hemocyte-like cells homozygous for the 2L+^a^ karyotype for a total of 6 samples. After sequencing, we obtained ~100 million unique mappable reads for each replicate. Non-duplicate *cis* read pairs accounted for >40% of total read pairs for all replicates, passing the QC metric for deep sequencing for Dovetail Genomics. Library statistics for Micro-C data are available in Additional File 3, Tables S11&S12. All Micro-C data is available at PRJNA1439337.

### Micro-C Analysis

#### Micro-C read processing and mapping

Read processing and mapping was performed based on the workflow developed by Dovetail Genomics (https://dovetail-analysis.readthedocs.io/en/latest/). Briefly, fastq files were aligned to the *A. gambiae* PEST reference genome (VectorBase-68_AgambiaePEST_Genome.fasta) or *A. coluzzii* MOPTI reference genome (VectorBase-68_AcoluzziiMOPTI_Genome.fasta) using BWA-MEM (v.0.7.17)^120^ with options −5SP -T0 -t16 for each replicate independently. Pairtools parse (v.1.0.3)^121^ with options --min-mapq 40 --walks-policy 5unique --max-inter-align-gap 30 --nproc-in 8 --nproc-out 8 was used to find ligation events. Parsed pairs were sorted and PCR duplicates were removed using pairtools sort with --nproc16 and pairtools dedup with --nproc-in 8 --nproc-out 8 --mark-dups, respectively. Pairtools split with --nproc-in 8 --nproc-out 8 generated a .pairs file and a .bam file, which was sorted and indexed using samtools (v1.20)^122^.

#### Generation of Contact Matrices

Contact matrices were generated using Juicer Tools (v1.22.01)^123^ with -Xmx48000m - Djava.awt.headless=true --threads 16. Contact matrices were visualized using Juicebox^124, 125^.

#### Topology feature discovery

TADs were called using hicexplorer hicFindTADs (v3.7.6)^70–72^ with default parameters on Knight-Ruiz (KR) normalized .cool files that were converted from .hic contact matrices of Ag55 samples mapped to PEST and Sua4.0 samples mapped to MOPTI. A resolution of 5kb was used for TAD calling. A/B compartments were assigned using fanc compartments (v0.9.28)^126^ with default parameters at 50kb resolution.

#### Comparing chromatin interaction differences between cell line samples

Significant (|log2FC|>1, p<0.05) differential interactions between Ag55 and Sua4.0 cell line samples were considered to occur over the 2La inversion if at least one anchor overlapped with the 2La inversion. A chi-square goodness-of-fit test was the statistical test performed to determine whether the number of interactions over the 2La inversion was enriched for the genomic length of the inversion as compared to the total length of chromosome arm 2L. The same definition and analysis were used for analysis of significant differential interactions involving chromosomal inversion 2Rbc. Plotting the genomic position of anchors involved in significantly different interactions (|log2FC|>1, p<0.05) was performed using R packages karyoploteR (v1.32.0)^127^ and GenomicRanges (v1.58.0)^128^.

#### Determining inversion karyotype of cell line samples using molecular assays

Genomic DNA was isolated from Sua4.0 (2La/2La hemocyte-like cells) and Ag55 (2L+^a^/2L+^a^ hemocyte-like cells). The 2La inversion karyotype of each cell line was confirmed using a previously published PCR assay for 2La molecular karyotyping^23^. The two cell lines were selected for their known alternate 2La inversion karyotypes. The karyotypes of known chromosome arm 2R inversions for which there are molecular assays (2Rj^60^, 2Rb^33^, 2Rc^37^, 2Ru^61^, and 2Rd^129^) were also determined for each cell line, confirming Micro-C results. Both Sua4.0 and Ag55 are 2R+^j^/2R+^j^, 2R+^u^/2R+^u^, and 2R+^d^/2R+^d^. They differ in 2Rb and 2Rc inversion karyotype where Sua4.0 are 2R+^b^/2R+^b^ and 2R+^c^/2R+^c^ while Ag55 are 2Rb/2Rb and 2Rc/2Rc (Additional File 1, Fig S1).

#### Using multiHiCcompare to identify interacting regions

MultiHiCcompare^62^ was used for both within-group replicate analysis and comparative analysis of Micro-C datasets. Both analyses used make_hicexp and cyclic_loess functions to normalize and identify chromatin interactions supported by all three biological replicates from each cell line while comparative analysis additionally used the hic_exactTest function to identify differentially interacting regions between cell lines. MultiHiCcompare was selected as the program of choice for its comprehensive, unbiased approach. Chromatin loops are defined as extrusions of DNA through the ring-shaped cohesion protein that are arrested by CTCF protein binding to CTCF binding sites on the DNA^130^. In general, chromatin loop callers identify regions of increased interaction frequency compared to background or neighboring regions, using either algorithms on the contact matrix counts or on the visual image created by contact matrices^130, 131^. Because chromatin loops are better understood in humans and mice and loop formation in insects may be slightly different, involving other CTCF-like insulator proteins like Beaf-32^70, 132, 133^, CP190^133, 134^, and Chromator^133, 134^, we found existing chromatin loop callers to be biased in their identification of interacting regions and produce a limited number of chromatin interactions. For example, the loop caller Mustache^135^, which identifies a higher number of published ChIA-PET and HiChIP loops as compared to other loop callers HiCCUPS and SIP, identified 648 interactions on chromosome arm 2L in 2L+^a^ cells at 5kb resolution. In contrast, multHiCcompare^62^ identified 309,139 interactions on chromosome arm 2L in 2L+^a^ cells at 5kb resolution. MultiHiCcompare identified 470 of 648 interactions that Mustache identified (72.5%), and the 178 interactions identified by Mustache that were not identified by multiHiCcompare all had average raw read counts ≤ 5, which multiHiCcompare considers low average read count and intentionally filters out. If the low read count filter is removed, the overlap of Mustache-identified interactions also identified by multiHiCcompare increases from 72.5% to 100%. In addition to identifying the same interactions as Mustache, multiHiCcompare has a number of other advantages including (1) it takes multiple biological replicates as input data to identify interacting regions, and (2) it does not implement a distance cut off (as evidenced in Fig1G). Given these advantages, we used multiHiCcompare for an agnostic identification of chromatin interactions.

For comparative analysis, three biological replicates of 2La samples were compared to three biological replicates of 2L+^a^ samples, both mapped to PEST (2L+^a^ reference genome) version Agam_P4 (VectorBase release 68)^67, 68^. Within-group replicate analysis was performed only on 2L+^a^ samples mapped to PEST to facilitate analysis. Contact matrices generated by Juicer Tools^123^ were converted into sparse files for each chromosome independently using straw (v0.1.0)^125^. R package multiHiCcompare (v1.24.0)^62^ was used to import the sparse files of Micro-C replicates at 5kb resolution, perform cyclic loess normalization, and, if applicable, detect interaction differences. The hicexp object was created using the default parameters, except for remove.regions = NULL because a non-human genome was used. Default parameters zero.p and Amin filtered out low interaction counts, thus only keeping interactions supported by all biological replicates. Zero.p=0.8 removed interactions in which >80% of samples had a raw interaction count of 0, and Amin=5 removed interactions in which the raw interaction counts across the biological replicates being analyzed averaged ≤5. Normalization was performed across the biological replicates using the cyclic_loess function with default parameters. For comparative analysis, the hic_exactTest function was used with default parameters. Differentially interacting regions were defined as regions where |log_2_foldchange| ≥1, log counts per million ≥0.5, and adjusted p-value≤0.05.

### Identifying differentially interacting regions over inversion breakpoints

#### Defining the 2La breakpoint

Positions of 2La inversion breakpoints in PEST version AgamP4 (VectorBase release 68) were determined using PCR primers for molecular karyotyping of 2La and 2L+^a^ chromosomes^23^. Primer DPCross5^23^ blasts to PEST positions 20,528,07220,528,094bp; therefore 20,528,072bp is used as the proximal breakpoint. Primer 27A2^23^ blasts to PEST positions 42,165,607–42,165,626bp; therefore 42,165,626bp is used as the distal breakpoint. All mapping was to the PEST 2L+^a^ reference genome. For visualization, the schematic of the 2La chromosome in Fig2B was shown on the MOPTI 2La reference genome background (version 2021–03-25, VectorBase release 68). MOPTI breakpoints were assigned to a region analogous to PEST based on the position of genes internal vs external to each inversion breakpoint. MOPTI position 86,510,662bp is used for the proximal breakpoint, and 107,557,222bp is used for the distal breakpoint.

#### Defining the 2Rbc breakpoint

2Rb inversion breakpoints were previously defined as 19,023,925bp and 26,758,676bp in PEST^32^. 2Rc inversion breakpoints were previously defined as 26,758,676bp and 31,488,544bp in PEST^36^. Because inversions 2Rb and 2Rc occur over adjacent regions, sharing a breakpoint, and the two inversions have been reported as being in almost perfect linkage disequilibrium^37^, here we treat inversion 2Rbc as one inversion and perform the same analyses as was done for the 2La inversion.

#### Defining differential interactions over inversion breakpoints containing enhancer-promoter pairs

28,273 differential interactions between 2La and 2L+^a^ samples on chromosome arm 2L were determined from multiHiCcompare exactTest^62^ (see above). Three filters were applied to these 2L differential interactions to find evidence of enhancer reciprocal rewiring associated with the 2La inversion: 1) the interaction had to occur over a 2La breakpoint, 2) one anchor had to contain at least 1 enhancer and the other anchor had to contain the promoter region (defined as the 1000bp region upstream of the protein coding gene) for at least one DEG, and 3) each enhancer and DEG promoter had to interact over a 2La breakpoint in both 2La and 2L+^a^ samples. For filtering criteria (1), an interaction was defined as occurring over an inversion breakpoint if the breakpoint coordinate fell between the outermost coordinates of the two interaction anchors. There were 153 differential interactions over the proximal breakpoint (using PEST position 20528073bp), and 183 differential interactions over the distal breakpoint (using PEST position 42165625bp). Seven interactions occur over both the proximal and distal breakpoint, i.e. the interaction spans across the entire inversion and both breakpoints. All 7 of these interactions are present in 2La samples and absent in 2L+^a^ samples; therefore, they are likely an artifact of mapping 2La samples to a 2L+^a^ reference genome. These seven interactions were not double counted to arrive at the 329 total unique differential interactions occurring over a 2La inversion breakpoint, and they were filtered out in the downstream criteria. For filtering criteria (2), an enhancer was defined as being detected in at least 2 of 3 replicates from a previously published STARR-seq experiment^49^, and DEGs were defined as having a |log_2_foldchange| ≥1 and adjusted p-value ≤0.05 from our RNA-seq data. Promoters of the DEGs were defined as the 1000bp region upstream of the protein coding gene as described by VEuPathDB^67, 68^. A bed file of enhancers detected in at least 2 of 3 replicates from a previously published STARR-seq experiment^49^ is found in Additional File 2. This list is inclusive of the previously published 3288 STARR-seq *Anopheles* enhancers^49^. Of the 329 total unique differential interactions occurring over a 2La inversion breakpoint, 17 interactions contained at least 1 enhancer and at least one DEG promoter in each anchor (Fig2A).

Because of our interest in identifying enhancer reciprocal rewiring, filtering criteria (3) required that each enhancer and DEG promoter interacted over a breakpoint in both 2La and 2L+^a^ cells. This resulted in 13 interactions that satisfied all criteria, involving 5 candidate enhancers that interact with the promoter region of four different genes in an inversion-specific manner (Fig2A). A table of all 329 differential interactions and their categorization are found in Additional File 3, Table S7.

#### Characterizing differential interactions by enhancer-promoter content

To categorize the types of differential interactions, for each interaction, we identified all DEG promoters, non-DEG promoters, and enhancers found in each of the two anchors comprising the interaction. All differential interactions were assigned to one of five distinct categories. If a DEG promoter and enhancer were in opposite anchors of an interaction, the interaction was classified as a (1) valid DEG enhancer-promoter pair. If a non-DEG promoter and enhancer were in opposite anchors of an interaction, the interaction was classified as a (2) valid non-DEG enhancer-promoter pair. If one anchor had both a promoter (DEG or non-DEG) and an enhancer while its partner anchor did not have a promoter nor an enhancer, the interaction was classified as (3) asymmetric. If one anchor had either an enhancer or a promoter while its partner anchor did not have a promoter nor an enhancer, the interaction was classified as a (4) single-element interaction. If neither anchor of an interaction contained a promoter nor an enhancer, the interaction was classified as (5) lacking annotation. A chi-squared goodness-of-fit test was performed for statistical comparison of interaction categorization between differential interactions over 2La inversion breakpoints and over 2Rbc inversion breakpoints.

### ATAC-seq/Differential ATAC-seq

#### ATAC-seq library preparation and sequencing

Two biological replicates each of Ag55 and Sua4.0 cells were submitted to ActiveMotif for ATAC-seq library preparation and sequencing. Briefly, 3.7–6.8×10^6 cells frozen in cryopreservation solution per replicate were lysed with a nonionic detergent to yield pure nuclei. Chromatin was fragmented and tagmented with sequence adapters using the Tn5 transposase for library generation. After purification, the library was amplified by PCR, then sequenced on an Illumina NextSeq platform to generate 72–98 million 2×51bp read pairs per sample. After sequencing, we obtained 31–50 million unique paired reads for each replicate. FriP (fraction of reads in peaks) scores were 56–67% per replicate, passing the >30% QC metric for ActiveMotif. Number of peaks per replicate was 55,000–64,000, passing the >50,000 peaks QC recommendation by ENCODE^136^. Library statistics for ATAC-seq data are available in Additional File 3, Table S13. All ATAC-seq data is available at PRJNA1439337.

#### ATAC-seq read processing and mapping

ActiveMotif performed ATAC-seq and data analysis, using the nf-core/atacseq pipeline^137^. Briefly, fastQC^114^ was used for quality control and Trim Galore^138^ was used for adapter trimming. Sequence reads were aligned to PEST using bowtie2^139^. Peak calling was performed by MACS2^140^ using --broad. DESeq2^63^ was used to identify statistically significant differential peaks after a pre-filtering step that kept only rows with at least 10 total reads. Differentially accessible regions were defined as having a |log2FC| ≥1 and FDR ≤0.05.

#### Comparing STARR-seq peaks with other published datasets

Published open chromatin and epigenetics marker information was extracted from previously published work, and STARR-seq peaks were compared to identify active enhancers as previously described^51^. Briefly, peaks within +/− 1500bp of a given STARR-seq peak were considered to be overlapping. Candidate STARR-seq peaks were compared to ATAC-seq data from 4a-3A hemocyte-like cells from our previous work^51^, FAIRE-seq data from 4a-3B hemocyte-like cells^51^, ChIP-seq data from blood-fed and *Plasmodium*-infected mosquitoes^16^, and ATAC-seq data from midgut and salivary gland tissues of *Plasmodium*-infected mosquitoes^17^.

#### Predicting enhancer-promoter interactions using PSYCHIC

PSYCHIC^69^ was run on a MacBook Pro using Python2.7 to enable python scripts to interact with MATLAB. PSYCHIC was run according to its GitHub instructions (https://github.com/dhkron/PSYCHIC). For input, PSYCHIC takes a Hi-C contact matrix, promoter bed file, and a series of other information including resolution of the contact matrix, interaction distance cutoff, chromosome name, and chromosome size. The output from PSYCHIC is a bed file of predicted enhancer-promoter interactions with coordinates of the putative enhancer, the name of its candidate target gene, distance of the gene to putative enhancer, FDR, p-value, number of expected interactions, and number of observed interactions. Using a p-value cutoff of ≤0.05, we identified putative enhancer-promoter pairs in our 2L+^a^ samples mapped to PEST 2L+^a^ reference genome version AgamP4 (VectorBase release 68) and our 2La samples mapped to MOPTI 2La reference genome version 2021-03-25 (VectorBase release 68). We filtered to identify enhancer-DEG promotor pairs occurring over a 2La breakpoint, with PEST positions 20,528,072bp as the proximal breakpoint and 42,165,626bp as the distal breakpoint and MOPTI positions 86,510,662bp as the proximal breakpoint and 107,557,222bp as the distal breakpoint, as described in above methods.

#### Calculating distance-normalized interaction frequencies to correct for distance-decay

Chromatin interactions are expected to decay with physical distance because regions that are physically closer will inherently interact more frequently. To evaluate whether chromatin interactions are enriched, one must first understand the null expectation for interactions given the distance between two anchors. To achieve this, we calculated a local null distribution of expected interaction frequency for each distance by performing linear regression analysis on log_10_-transformed distance and log_10_-transformed average interaction frequency for all interactions with a given anchor. With a null distribution, we were able to calculate residuals, a z-score, and a corresponding two-tailed p-value to indicate how frequent or infrequent an interaction is relative to other interactions of that same distance. A Shapiro-Wilk test was performed to confirm a normal distribution of residuals prior to calculating a p-value for each z-score. If the residuals failed the Shapiro-Wilk test for normality, a non-parametric robust z-score was calculated prior to calculating a p-value for each robust z-score. A robust z-score uses median and median absolute deviation instead of mean and standard deviation, respectively. A z-score >0 indicates enrichment while a z-score <0 indicates underrepresentation. A z-score equaling 0 indicates an interaction frequency as expected for its distance as compared to the local null distribution of interaction frequencies. We classified degree of enrichment/underrepresentation based on the number of standard deviations away from the expected interaction frequency. Greater than 1.96 standard deviations (|z-score|>1.96) equates to a p-value≤0.05, so these interactions were considered significantly enriched (if positive) or underrepresented (if negative). Between 1 and 1.96 standard deviations (1≤|z-score|<1.96) was considered moderately enriched (if positive) or underrepresented (if negative). Between 0 and 1 standard deviations (0<|z-score|<1) was considered mildly enriched (if positive) or underrepresented (if negative). We do not expect all biologically relevant interactions to be significantly enriched given that genes can be regulated by multiple enhancers and a single enhancer can regulate multiple genes^49, 74^.These analytical and statistical methods enabled prioritization of enhancers or promoters for future investigation based on the z-score/p-value of their interaction. As above, an enhancer was defined as being detected in at least 2 of 3 replicates from a previously published STARR-seq experiment^49^. The 1000bp region upstream of the protein coding gene as described by VEuPathDB^67, 68^ is used as a proxy for a gene’s promoter. For visualization in Integrative Genomics Viewer (IGV)^141^, z-scores were plotted as a heatmap, and the colorimetric scale in Figure 3A, Figure 3C, and Figure 4 was set to red for a z-score ≥2 (p-value≤0.0455), white for a z-score = 0 (p-value = 1), and blue for a z-score ≤−2 (p-value≤0.0455).

### Measurement of enhancer activity by luciferase reporter assays

#### Cloning of candidate enhancers

As previously described^49, 50^, candidate enhancer regions were PCR amplified from genomic DNA isolated from either Sua4.0 (2La) or Ag55 (2L+^a^) cells. PCR reactions consisted of 10ng template DNA, 1X High-Fidelity PCR Master Mix with HF (Thermo), and 0.5uM of each forward and reverse primer. Cycling conditions were initial denaturation at 98C for 30s, 30 cycles of a 10s denaturation at 98C, 55–62C annealing temperature for 30s, and a 45s extension at 72C, followed by a final 10min extension at 72C. Resulting PCR products were either restriction enzyme cloned or Gateway cloned into a Firefly luciferase reporter vector pGL-Gateway-DSCP (AddGene, vector ID# 71506). Ligation products were transformed into OneShot OmniMax 2T1 Phage-Resistant Cells (Invitrogen), then grown overnight, plasmid purified, and sequenced. Amplification primers for candidate enhancers can be found in Additional File 3, Table S14. Note these primers are designed to be inclusive of published enhancers^49^; therefore, the amplicons are slightly larger than the STARR-seq published enhancer. Sequences of cloned alleles can be found in Additional File 4.

#### Lipid based transfection and luciferase reporter assays

*A. coluzzii* Sua4.0 or Ag55 cells were seeded at 2.5×10^4 cells/well in 65ul in a 96 well plate. Cells were agitated on a MixMate (Eppendorf) for 30s at 300rpm for even distribution. Following a 24hr incubation period, lipid-based transfections were performed using Lipofectamine3000 (Invitrogen). Cells were transfected with two plasmids (1) the pGL-Gateway-DSCP (described above) carrying a single amplified fragment of the candidate enhancer upstream of a firefly luciferase gene and (2) a renilla control vector pRL-ubi-63E (AddGene, #74280). Plasmids were transfected at a ratio of 1:5 (renilla:firefly). Plates were agitated again for 30s at 300rpm on a MixMate (Eppendorf) and incubated for 24h at 27C. The Dual-Glo Luciferase Assay System (Promega) was used for luciferase assays, according to supplier instructions. Measurements were recorded on the GloMax Discover (Promega) at 25°C. All test plates contained cells transfected with control plasmid constructs: a negative control fragment, which was a size-matched fragment within intron 1 of AGAP007058 (DLX), and a highly active positive control enhancer peak nearby AGAP008980^111^. All samples were run in 6-fold technical replication within a single plate and across at least two independent plates for at least two biological replicates. Firefly luciferase measurements were normalized to renilla measurements from the same well. These measurements were then expressed relative to the firefly/renilla mean for the negative control on the same plate. A one-way ANOVA followed by Tukey’s multiple comparison test was used to compare the activities of enhancer alleles assayed across different cellular backgrounds.

## Supplementary Files

This is a list of supplementary files associated with this preprint. Click to download.

- Tables.docx
- AdditionalFile1.pdf
- AdditionalFile2.bed
- AdditionalFile3.xlsx
- AdditionalFile4.fasta

## Figures and Tables

**Figure 1.**
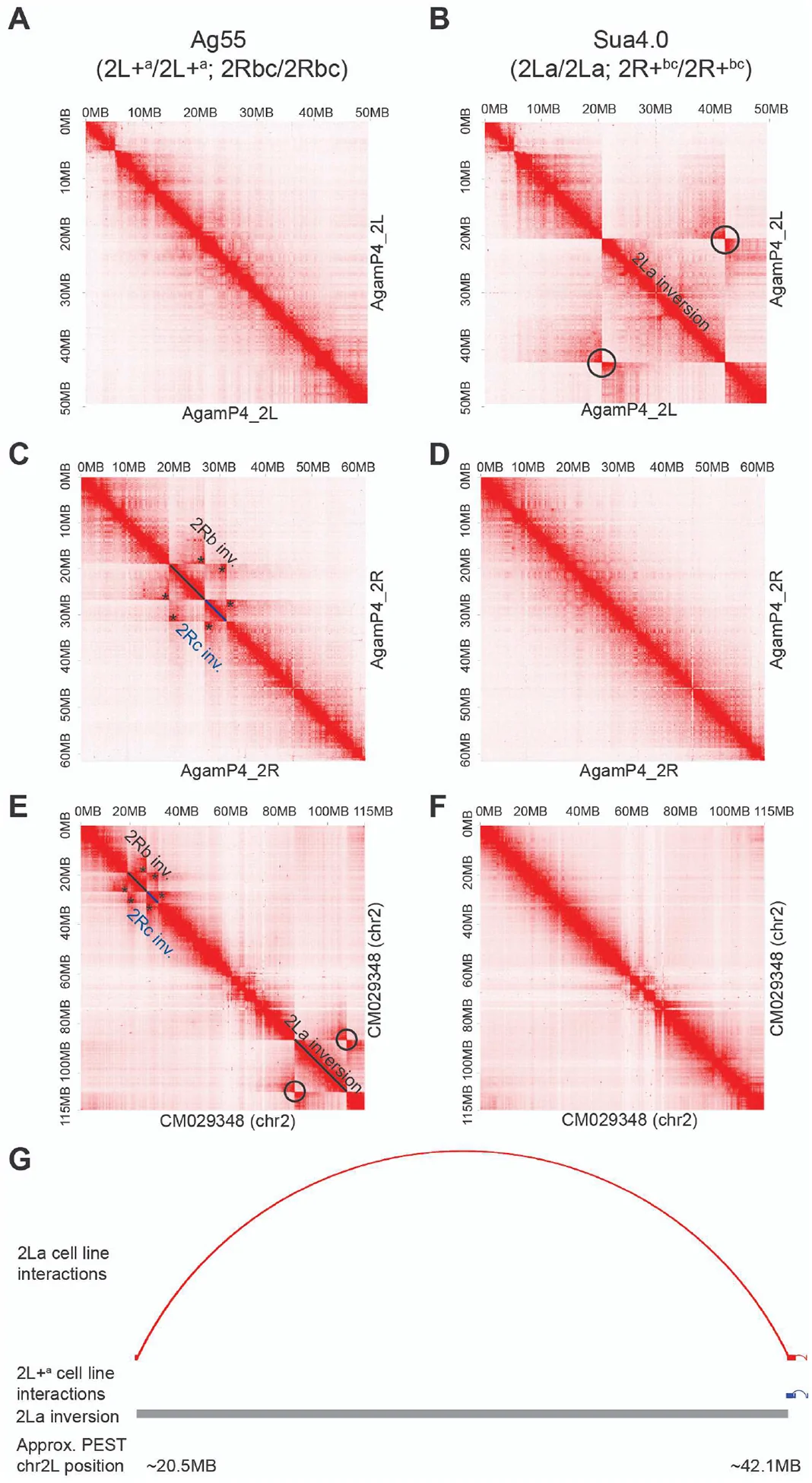
Proximity ligation characterization of hemocyte-like cell lines fixed for alternate 2La inversion karyotypes. **A-B)** Chromosome arm 2L contact matrices of cell line Ag55 fixed for 2L+^a^ (**A**) or cell line Sua4.0 fixed for 2La (**B**) mapped to the *A. gambiae* 2L+^a^ PEST reference genome. In B, the butterfly pattern (circled) indicates the 2La inversion (labeled line) when a sample of one inversion karyotype (2La) is mapped to a reference genome of the alternate karyotype (2L+^a^). **C-D)** Chromosome arm 2R contact matrices of cell line Ag55 fixed for 2L+^a^ (**C**) or cell line Sua4.0 fixed for 2La (**D**) mapped to the 2L+^a^ PEST reference genome. In C, the asterisks mark irregular interaction structure, and the labeled lines show the 2Rb and 2Rc inversions. **E-F)** Chromosome 2 contact matrices of cell line Ag55 fixed for 2L+^a^ (**E**) or cell line Sua4.0 fixed for 2La (**F**) mapped to the *A. coluzzii* 2La MOPTI reference genome. Chromosome CM029348 in the MOPTI genome is chromosome 2 with both L and R arms. In E, the asterisks mark irregular interaction structure, the 2La inversion butterfly pattern is circled, and the labeled lines show the 2Rb, 2Rc, and 2La inversions. MOPTI is non-colinear to PEST regarding the 2La inversion, evidenced by the 2La butterfly pattern in 2La samples when mapped to PEST 2L+^a^ reference (**B**) but in 2L+^a^ samples when mapped to MOPTI 2La reference (**E**). MOPTI is co-linear to PEST regarding the 2Rb and 2Rc inversions, evidenced by the irregular interaction structure in both PEST- (**C**) and MOPTI-mappings (**E**) of cell line Ag55 fixed for 2La^+^. **G)** Interactions (shown as arcs) from multiHiCcompare within-group replicate analysis of the 5kb region with PEST positions 42260000–42265000bp (94,375bp outside the 2La inversion) that are present in the 2La fixed cell line Sua4.0 (top, red) and the 2L+^a^ fixed cell line Ag55 (bottom, blue) mapped to the 2L+^a^ PEST reference genome. The ~21MB red arc depicts interactions between physically proximal regions in the 2La karyotype, but here when mapped to the 2L+^a^ reference genome, the interaction spans ~21MB (the length of the 2La inversion).

**Figure 2.**
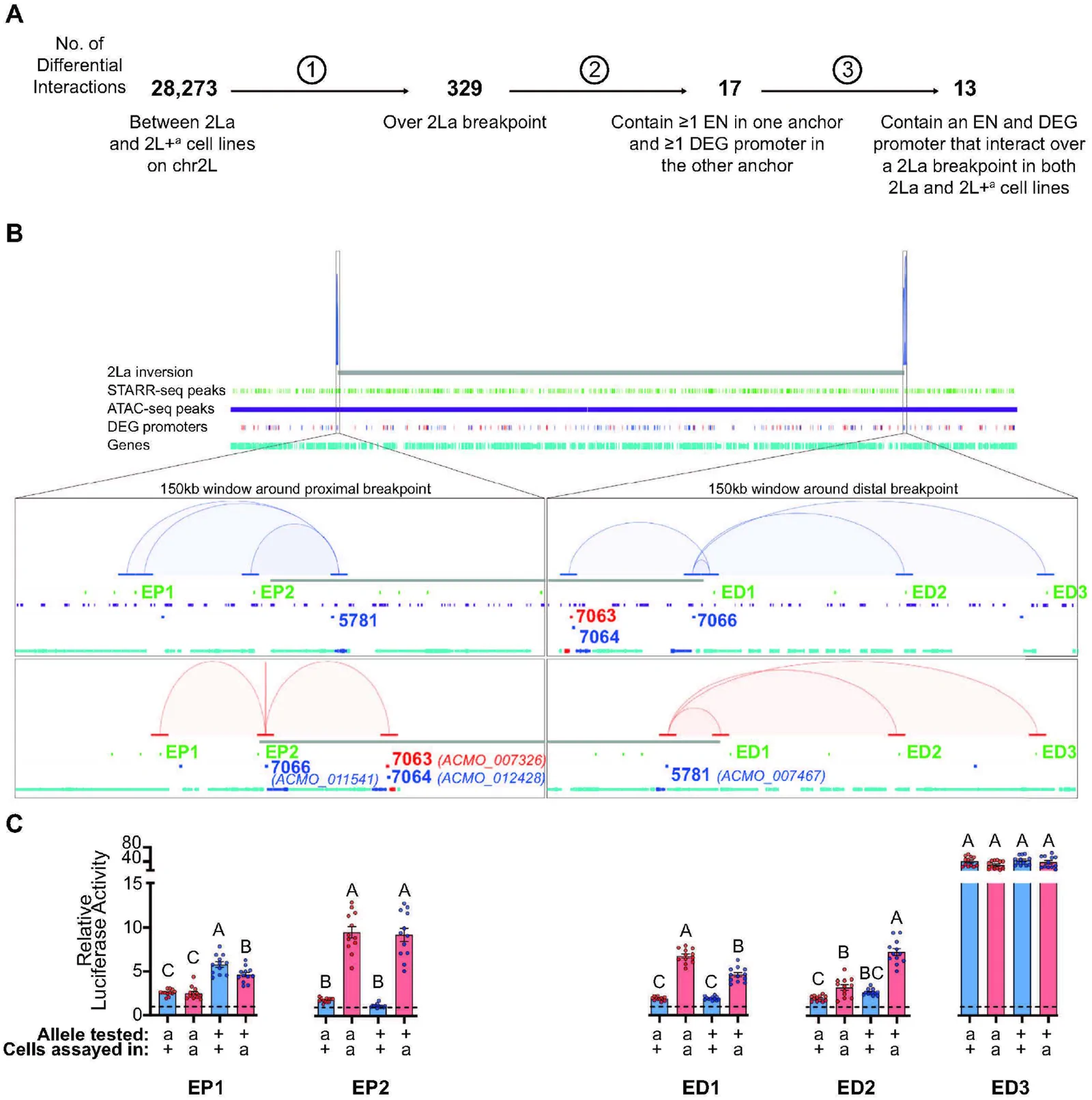
Proximity ligation uncovers novel enhancer rewiring over 2La inversion breakpoints. **A)** Flow chart showing the filtering criteria of differential interactions on chromosome arm 2L between 2La and 2L+^a^ cell line samples to identify interactions of interest. Differentially interacting regions between 2La and 2L+^a^ samples were determined using multiHiCcompare comparative analysis of 5kb resolution Micro-C data. Filter (1) required that differential interactions occur over a 2La inversion breakpoint (PEST: 20,528,073bp, proximal; 42,165,625bp, distal). Filter (2) required that one anchor of the interaction contained at least one enhancer (EN), and the other anchor contained the promoter region of at least one differentially expressed gene (DEG). To identify evidence of enhancer reciprocal rewiring, filter (3) required that each enhancer and promoter had to interact over a 2La breakpoint in both 2La and 2L+^a^ samples. **B)** Schematics depicting the 13 differential chromatin interactions occurring over a 2La inversion breakpoint, involving enhancers and promoters that differentially interact in an inversion-specific manner. Upper image is a 30MB window around the 2La inversion (gray bar) with distributions of STARR-seq peaks (green), ATAC-seq peaks (purple), promoter regions of DEGs (blue for upregulated in 2L+^a^ sample, red for upregulated in 2La sample), and genes (teal). Black boxes at both ends of the inversion highlight the regions where chromatin interactions occur over the breakpoint, and these regions are shown at high resolution below. Lower images show 2L+^a^ interactions (in blue) and 2La interactions (in red) in 150kb windows around the proximal (left) and distal (right) breakpoints of the 2La inversion. For illustration purposes, the 2La interactions (red) are shown on the MOPTI (2La reference genome) background to emphasize that in the 2La karyotype, genes AGAP007066, AGAP007064, and AGAP007063 are near the proximal breakpoint and gene AGAP005781 is near the distal breakpoint. Horizontal blocks connected by arcs represent 5kb genomic regions and an interaction between the two 5kb anchor regions. The vertical red “line” at EP2 is a narrow arc indicating that region interacting with itself, which contains both EP2 and gene AGAP007066. The gray bar indicates the extent of the 2La inversion. Genomic elements in the tracks below the 2La inversion (gray bar) are colored as in the upper image: STARR-seq peaks (green), ATAC-seq peaks (purple), promoter regions of DEGs (blue for upregulated in 2L+^a^ sample, red for upregulated in 2La sample), and genes (teal). The labeled promoters (5781, 7063, 7064, 7066) meet the filtering criteria from panel A. Note: the leading “AGAP00” for the labeled gene promoters has been omitted. Enhancer rewiring is evidenced by a number of interactions: in 2L+^a^ cells, EP1 and EP2 interact with 5781 while in 2La cells, they interact with 7066, and EP2 additionally interacts with 7064 and 7063. In 2L+^a^ cells, ED1, ED2, and ED3 interact with 7066, and ED1 additionally interacts with 7064 and 7063 while in 2La cells, they interact with 5781. **C)** All five candidate enhancers involved in chromatin interactions spanning an inversion breakpoint were tested for enhancer activity with a luciferase assay. Candidate enhancer alleles were cloned from 2L+^a^ cells or 2La cells into luciferase reporter plasmid pGL_Gateway-DSCP, and enhancer activity was tested using a dual glo luciferase assay system. Enhancer activity of 2L+^a^ alleles (blue dots) or 2La alleles (red dots) were tested in 2L+^a^ homokaryotic cells (blue bars) or 2La homokaryotic cells (red bars) to quantify luciferase activity above background, defined as negative control (dotted line, y=1) as used previously^111^. Y-axis indicates the relative luciferase activity for each measurement, expressed as firefly luciferase corrected to the renilla luciferase internal control value, and normalized for the value of the negative control. All samples were run in 6-fold technical replication, across two biological replicates. A one-way ANOVA followed by Tukey’s multiple comparison test was used to compare allele and cellular background combinations to each other for each enhancer. Bars that share a letter are not statistically different while bars that do not share a letter are statistically different (p<0.05). The most biologically relevant cases are the innermost bars in which enhancer alleles are tested in their respective cell lines. In these cases, the EP1 2L+^a^ allele has significantly higher activity, the EP2 and ED1 2La alleles display significantly higher activity, and the enhancer activities of ED2 and ED3 do not differ between 2L+^a^ and 2La alleles when assayed in their respective cell lines. Error bars represent +/− 1 standard deviation.

**Figure 3.**
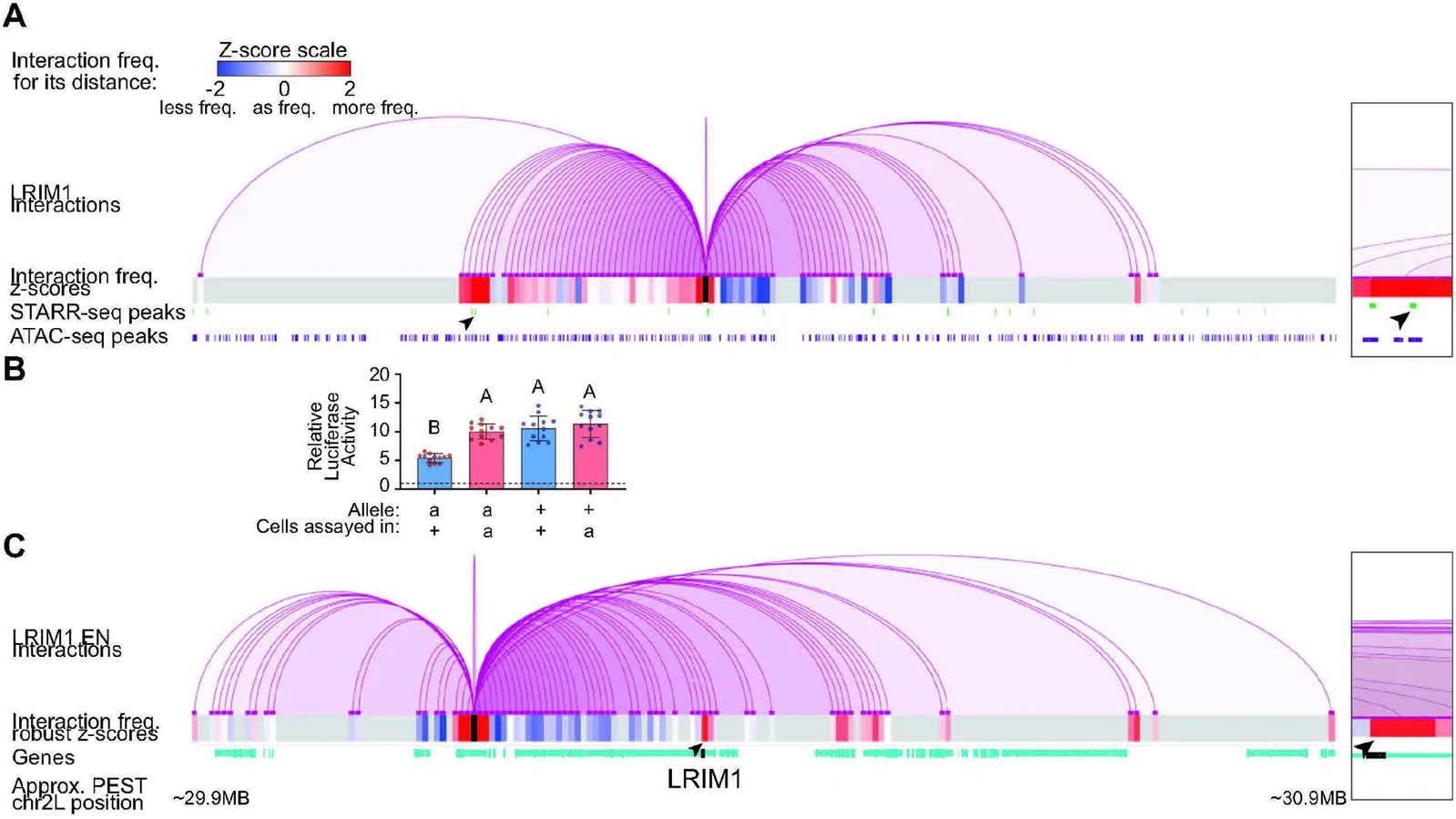
Micro-C enables prioritization of a candidate enhancer located ~180kb downstream of LRIM1 promoter. **A)** Interactions (purple arcs and their associated 5kb anchors) anchored on the LRIM1 promoter region (AGAP006348, black box) were generated from multiHiCcompare within-group replicate analysis of 5kb resolution 2L+^a^ Micro-C contact matrices. Interaction frequency z-scores were calculated as in Fig3, and the z-score scale is shown, where red ≥2 (interaction is more frequent than expected for its distance), white = 0 (interaction frequency is as expected for its distance), and blue ≤−2 (interaction is less frequent than expected for its distance). The arrowhead points to the STARR-seq peak (AgamP4_2L:30142958–30143475) that is a novel candidate LRIM1 enhancer (z-score 2.16, p-value <0.05). This enhancer overlaps with an ATAC-seq peak (dark purple) and is marked by the histone mark H3K27ac^16^, suggesting it is an active enhancer. **B)** This novel candidate LRIM1 enhancer sequence was cloned from 2L+^a^ cells and 2La cells and enhancer activity was tested in both cellular backgrounds using a luciferase assay. Y-axis indicates the relative luciferase activity for each measurement, expressed as firefly luciferase corrected to the renilla luciferase internal control value, and normalized for the value of the negative control (dotted line, y=1). All samples were run in 6-fold technical replication, across two biological replicates. A one-way ANOVA followed by Tukey’s multiple comparison test was used to compare allele and cellular background combinations to each other. Bars that share a letter are not statistically different while bars that do not share a letter are statistically different (p<0.05). The most biologically relevant comparison is between the innermost bars comparing alleles within their respective cell lines, which are not significantly different. Error bars represent +/− 1 standard deviation. **C)** A reciprocal analysis instead anchored on the newly identified LRIM1 enhancer (AgamP4_2L:30142958–30143475) indicates the interaction between the candidate LRIM1 enhancer (black box) and the LRIM1 promoter region (arrowhead) occurs more frequently than expected for its distance (robust z-score 2.24, p<0.05). Apart from the regions immediately proximal to the enhancer-containing anchor, the LRIM1 promoter-containing anchor has the highest z-score.

**Figure 4.**
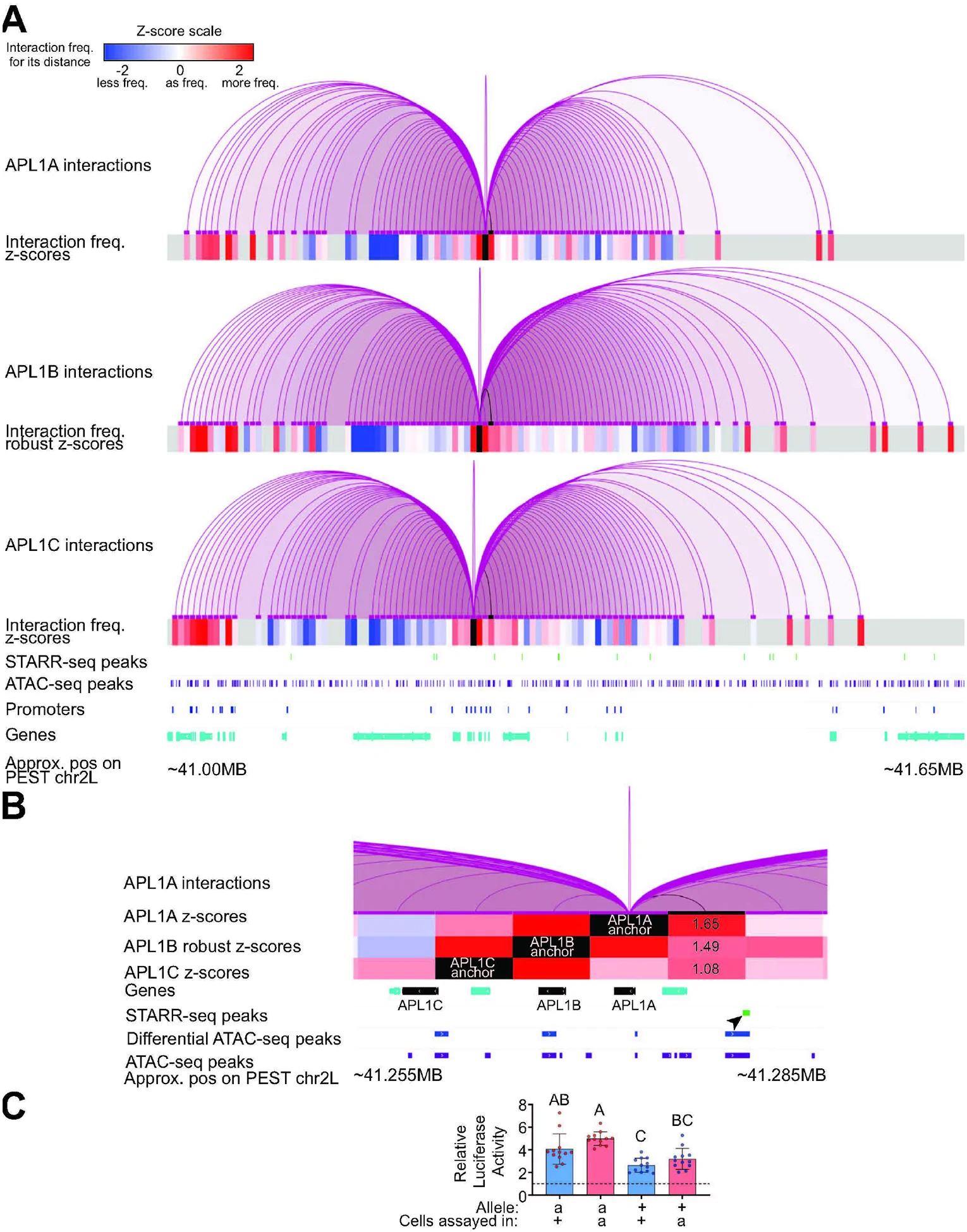
Novel analysis of Micro-C data identifies common enhancer for APL1A, APL1B, and APL1C. **A)** Schematic of chromatin interactions anchored on the promoter region of APL1A (top), APL1B (middle), and APL1C (bottom) and their distance-normalized interaction frequency z-scores (or robust z-scores if residuals were not normally distributed). Interactions (purple arcs and their associated 5kb anchors) for each promoter region (black vertical box), as defined by the 1000bp region upstream of a given protein coding gene, were generated from multiHiCcompare within-group replicate analysis of 5kb resolution 2L+^a^ Micro-C contact matrices. For each interaction, residual interaction frequences, defined as observed minus expected interaction frequency for a given distance, were standardized to z-scores. The z-score quantifies how frequent or infrequent an interaction is, corrected for its distance. Colored vertical boxes indicate z-score where red ≥2 (interaction is more frequent than expected for its distance), white = 0 (interaction frequency is as expected for its distance), and blue ≤−2 (interaction is less frequent than expected for its distance). Below the interactions, STARR-seq identified candidate enhancers are shown in green, ATAC-seq peaks are shown in dark purple, promoters are shown in navy, and genes are shown in teal. Ranking each gene’s interactions by highest z-score reveals that a common enhancer (AgamP4_2L: 41279774–41280274; APL1A z-score=1.65, p=0.10; APL1B robust z-score=1.49, p=0.14; APL1C z-score=1.08, p=0.28) is in the most frequent enhancer-containing interaction for all three genes. The interaction between each promoter region and the common enhancer is shown with a black arc and black anchor. **B)** An enlarged schematic focused on the 5kb region containing the common enhancer (green box and black arrowhead). Arcs are only shown for APL1A and interaction frequency z-scores for APL1A, B, and C are shown in the heatmap below the arcs. The z-scores for the common enhancer-containing regions are labeled within the region. The common enhancer not only overlaps with an ATAC-seq peak (dark purple) but also with a differential ATAC-seq peak that is more accessible in 2L+^a^ cells (blue). Red would indicate more accessible in 2La samples, but no such region is in the selected window. **C)** The candidate common APL1 enhancer sequence was cloned from 2L+^a^ cells and 2La cells, and enhancer activity was tested in both cellular backgrounds using a luciferase assay. Y-axis indicates the relative luciferase activity for each measurement, expressed as firefly luciferase corrected to the renilla luciferase internal control value, and normalized for the value of the negative control (dotted line, y=1). A one-way ANOVA followed by Tukey’s multiple comparison test was used to compare allele and cellular background combinations to each other. Bars that share a letter are not statistically different while bars that do not share a letter are statistically different (p<0.05). The most biologically relevant comparison is between the innermost bars comparing alleles within their respective cell lines, where the 2La allele displays significantly higher activity than the 2L+^a^ allele. Error bars represent +/− 1 standard deviation.

**Figure 5.**
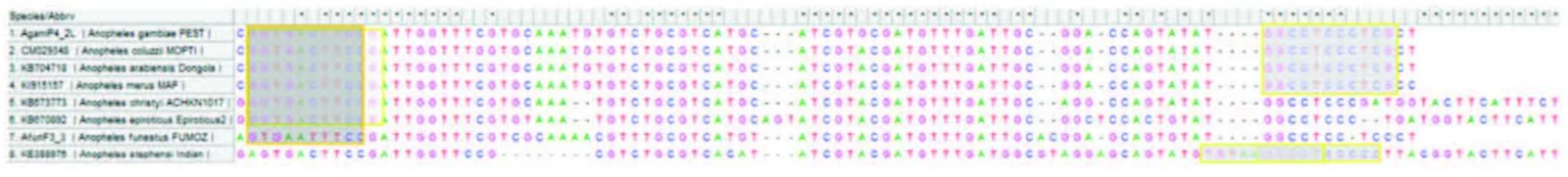
Alignment of the conserved region within the newly identified regulatory region of LRIM1. Sequences with significant (p<0.01) similarity to the Relish binding site at the 5’ and 3’ end of the sequence are shown in yellow bounded light gray shaded boxes. Sequences with significant similarity to the Dif binding site at the 5’ end of the sequence are outlined in orange and shaded in dark gray. At the 5’ end of the sequence, the Relish and Dif similar sites overlap for all species other than *A. funestus*, which only displays similarity to the Dif binding site. At the 3’ end of the sequence, *A. stephensi* and the members of the species complex have significant similarity to the Relish TFBS.

**Table 1.** The same enhancers are involved in enhancer reciprocal rewiring with nonDEGs as with DEGs. Gene functions are as follows: AGAP005781 is a glycine C-acetyltransferase. AGAP029526 is an unspecified product. AGAP007066 is tRNA_specific adenosine deaminase 3. AGAP007068 is RNA finger protein 113A. AGAP007065 is transcription factor CP2 and related proteins. AGAP007064 is an unspecified product. AGAP007063 is origin recognition complex subunit 4. AGAP007062 is ecdysis-triggering hormone.

**Table 2.** Additional differential enhancer-promoter interactions across 2La inversion breakpoints (not including the 13 differential interactions already characterized). N/A means the enhancer does not have an interaction with a promoter over a 2La breakpoint. For assessment, newly created means an enhancer-promoter interaction is not present over a breakpoint in the ancestral karyotype (2La) but is present in the derived karyotype (2L+^a^). Abolished means an enhancer-promoter interaction is present over a breakpoint in the ancestral karyotype (2La) but no longer present in the derived karyotype (2L+^a^). Re-wiring means an enhancer has different target genes over inversion breakpoints in the ancestral (2La) vs derived (2L+^a^) karyotypes; however, the interactions between enhancers and target genes are not interchanged as was the case for the 13 interactions identified in enhancer reciprocal rewiring. Gene names/function can be found in Additional File 3, Table S3.

**Table 3.** Differential enhancer-promoter interactions across 2Rbc inversion breakpoints. The same definitions for assessment are used as for Table 2; 2R+^bc^ was considered ancestral while 2Rbc was considered derived. Gene names/function can be found in Additional File 3, Table S3.

**Table 4.** Candidate target genes for enhancers characterized in Zmarlak-Feher et al. 2025 based on highest interaction frequency from distance-decay correction analysis. The top candidate target gene(s) were determined for the enhancers characterized in Zmarlak-Feher et al. 2025 by identifying the genes in the promoter-containing interactions with the highest interaction frequency z-score/robust z-score. Distances between candidate target gene and enhancer ranged from ~4–564kb.

## Data Availability

The data generated and analyzed during the current study are available in PRJNA1439337.

## ABBREVIATIONS

DEG: differentially expressed gene
DIR: differentially interacting region
IGV: Integrative Genomics Viewer
TFBS: transcription factor binding site
TAD: topologically associating domain
